# All-Atom Simulations Reveal a Key Interaction Network in the HLA-E/NKG2A/CD94 Immune Complex Fine-Tuned by the Nonameric Peptide

**DOI:** 10.1021/acs.jcim.1c00414

**Published:** 2021-07-01

**Authors:** Eva Prašnikar, Andrej Perdih, Jure Borišek

**Affiliations:** † National Institute of Chemistry, Hajdrihova 19, 1000 Ljubljana, Slovenia; ‡ Faculty of Pharmacy, University of Ljubljana, Aškerčeva 7, 1000 Ljubljana, Slovenia; § Graduate School of Biomedicine, Faculty of Medicine, University of Ljubljana, Vrazov trg 2, 1000 Ljubljana, Slovenia

## Abstract

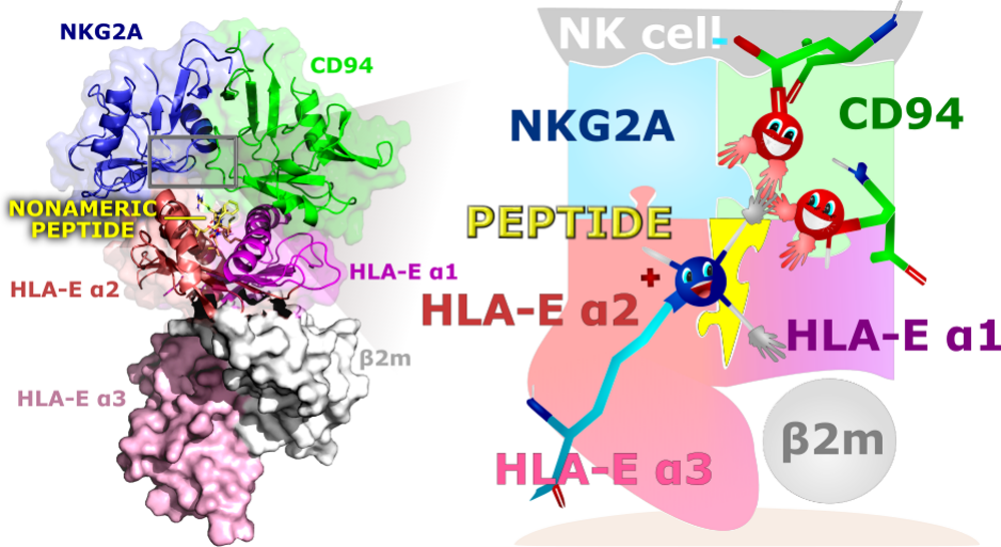

Natural killer (NK) cells, an important part of the innate immune system, can clear a wide variety of pathological challenges, including tumor, senescent, and virally infected cells. They express various activating and inhibitory receptors on their surface, and the balance of interactions between them and specific ligands displayed on the surface of target cells is critical for NK cell cytolytic function and target cell protection. The CD94/NKG2A heterodimer is one of the inhibitory receptors that interacts with its trimeric ligand consisting of HLA-E, β2m, and a nonameric peptide. Here, multi-microsecond-long all-atom molecular dynamics simulations of eight immune complexes elucidate the subtleties of receptor (NKG2A/CD94)–ligand (HLA-E/β2m/peptide) molecular recognition that mediate the NK cell protection from a geometric and energetic perspective. We identify key differences in the interactions between the receptor and ligand complexes, which are via an entangled network of hydrogen bonds fine-tuned by the ligand-specific nonameric peptide. We further reveal that the receptor protein NKG2A regulates the NK cell activity, while its CD94 partner forms the majority of the energetically important interactions with the ligand. This knowledge rationalizes the atomistic details of the fundamental NK cell protection mechanism and may enable a variety of opportunities in rational-based drug discovery for diverse pathologies including viral infections and cancer and elimination of senescent cells associated with potential treatment of many age-related diseases.

## Introduction

Natural killer (NK) cells are a subpopulation of lymphocytes, an important part of the innate immune system, that respond quickly without priming or preactivation to a wide variety of pathological challenges such as virally infected and tumor cells.^1^ They act through various molecule-specific receptors expressed on their surface, either via antibody-dependent or natural cytotoxicity.^2^ In the latter case, the balance between interactions with inhibitory and activating receptors is crucial for the cytolytic function of the NK cells. Externally, they present various inhibitory (e.g., CD94/NKG2A, -B, KIR2DL, and KIR3DL) or activating (e.g., NKG2D/NKG2D, KIR2DS, CD94/NKG2C, -E, and -H) receptors^3,4^ that allow them to directly recognize ligands (proteins) expressed on the target cells and determine their fate. Healthy cells tend to present MHC class I molecules, which are ligands for the inhibitory receptors and mediate protection from the NK cell clearance activity. On the other hand, cellular stress (e.g., DNA damage response, senescence program, tumor expression, viral infection, etc.) leads to the upregulation of expression of ligands for activating receptors (e.g., MIC-A/-B and ULBPs) or downregulation of normally present MHC class I molecules, shifting the balance in favor of NK cell activation and elimination of compromised cells.^2,5^

Several receptor families for the MHC class I molecules presented on the NK cells have been identified, including killer cell Ig-like receptors, immunoglobulin-like transcripts, and the C-type lectin family, which consists of a heterodimer of the CD94 protein and a representative of the NKG2 family molecules (NKG2-A/B, -C, -E, -H, and -F), except NKG2D, which exists as a homodimer. While the ligands for the former two receptor families are (Ia) HLA class I molecules and the non-classical HLA-G, the ligand for most CD94/NKG2 heterodimers is the non-classical (Ib) glycoprotein HLA-E.^2^ HLA-E is encoded by MHC HLA loci and is exhibiting a low level of polymorphic occurrences. Two nearly equally presented^6^ functional HLA-E alleles, HLA-E^R^ (HLA-E*01:01) and HLA-E^G^ (HLA-E*01:03),^6,7^ which differ in one amino acid at the position 107, Arg or Gly, respectively, account for almost 100% of HLA-E in the human population. Both alleles can be beneficial in certain settings as *01:01 allele is more favorable in cancer diseases and *01:03 is more favorable in bacterial infections, bone marrow transplantation, and periodic abortions.^6^ For cell surface expression of HLA-E, a trimeric complex composed of HLA-E, its light chain beta-2 microglobulin (β2m), and the specific nonameric peptide derived from signal sequences of other HLA class I molecules (e.g., HLA-A, -B, -C, and -G) must be established.^8,9^ The array of suitable nonameric peptides is restricted, and only a handful of them allow effective recognition of the HLA-E complex expressed on the cell surface by the receptor presented by the NK cells. This leads to either the subsequent death of the target cell or its protection from elimination.^10−16^ Of all the peptides studied to date, the HLA-G leader sequence proved to be the most effective mediator.^11,13^

Among the various NK cell receptors, the inhibitory CD94/NKG2A receptor complex, which upon activation enables cell protection, is the one with the highest micromolar affinity for the HLA-E complex. In fact, NKG2A holds a sixfold higher affinity for HLA-E compared to the activating receptor containing NKG2C.^13^

The implication of NK cells and their activating/inhibitory receptors has also recently been linked to aging, a process that affects the vast majority of living organisms and appears to be the major risk factor for the development of several age-related diseases, including diabetes,^17^ idiopathic pulmonary fibrosis,^18^ and cancer.^19^ Cellular senescence, a state of irreversible cell cycle arrest in response to cellular damage, has been identified as one of the key features of human aging that can be targeted to reduce the negative health effects of aging.^20,21^ At least three ligands for either activating or inhibitory NK cell receptors have been reported to be present on the senescent cells. With increasing age, also, more abundant expression of the NKG2A receptor ligand HLA-E on senescent cells derived from human skin was reported. Blocking the interaction of HLA-E and NKG2A has been shown to enhance the immune response against senescent cells *in vitro*, suggesting a potentially new strategy to eliminate senescent cells,^22^ thereby reducing their adverse health effects. Increased expression of the inhibitory NKG2A receptor associated with NK cell exhaustion was also detected in patients with severe COVID-19.^23^

Several crystal structures of various compositions of HLA-E/β2m/peptide/NKG2A/CD94 immune complex systems^7,16,24−26^ and mutagenesis studies^26,27^ have clarified the molecular details of the receptor–ligand interactions, while binding and cytotoxicity assays^10,25,28^ revealed the role of specific nonameric peptide residues. However, their mechanistic details and the intricacies of the small peptide-mediated NK cell action still remain elusive.

Here, we performed all-atom molecular dynamics (MD) simulations of several immune complexes of the HLA-E/β2m/NKG2A/CD94 system with nonameric peptides to elucidate the mechanistic details guiding the receptor NKG2A/CD94 molecular recognition of the ligand HLA-E/β2m/peptide at the atomistic level. We identified a hydrogen bonding network between CD94 and NKG2A that occurred more frequently in complexes with nonameric peptides, which effectively mediates the receptor–ligand recognition, allowing successful protection from NK cell elimination. In addition, we coupled these results with the analysis of the system internal dynamics and free energy calculations to provide an extensive picture of this complex molecular recognition event.

## Methods

### Structural Models

Eight different models were built based on the crystal structure of the extracellular domains of human CD94/NKG2A in complex with the extracellular domain of HLA-E [light (β2m) and heavy chain (α1, α2, and α3 subdomains)] and the leader peptide of the HLA class I histocompatibility antigen, alpha chain G, solved at a resolution of 3.4 Å (PDB entry: 3CDG).^24^ In our simulations, we took into account only the resolved extracellular domains of proteins CD94 (residues 57–179), NKG2A (residues 113–232), HLA-E (2–274), and β2m deprived of its signal peptide (residues 21–119), for which structural data are available. The CD94, NKG2A, and HLA-E proteins further consist of cytoplasmic and transmembrane domains, which were not considered in this study. The first model (i) comprises HLA-E, β2m, NKG2A, and CD94 proteins and the G nonameric peptide (sequence: VMAPRTLFL), hereafter referred to as **COM^+^_G_** (Supporting Information files COMg_structure.pdb and parameter file COMg_structure.top). The second model (ii) **^allele^COM^+^_G_** represents the allelic variant HLA-E*01:03, with the 107R > G (residue 107 coordinates taken from PDB entry 6GH1).^25^

Next, models differ from **COM^+^_G_** in the sequence of the nonameric peptide, a key element for a successful recognition of HLA-E by NKG2A/CD94 receptors located on NK cells. Namely, the complex with the HLA-B7 peptide (VMAPRTVLL) represents the third model (iii) **COM^+^**_**B7**_ (peptide taken from PDB entry 1KPR).^7^ Next, the model containing the Hsp60sp signal peptide (sequence: QMRPVSRVL), referred to as (iv) **COM^–^**_**Hsp60sp**_, was obtained by introducing several mutations into peptide G of model **COM^+^_G_** [sequence: (V > Q)M(A > R)P(R > V) (T > S) (L > R) (F > V)L]. Mutation R5V of peptide B7 [sequence: VMAP(R > V)TVLL] introduced in model **COM^+^**_**B7**_ resulted in model (v) **COM**^–^_**B7_R5V**_. Two additional models (vi) **COM**^**∼**^_**B27**_ and (vii) **COM**^**∼**^_**Cw7**_ with experimentally inconclusive NK cell protection were built, with model **COM**^**∼**^_**B27**_ containing the leader sequence of HLA-B27 (peptide taken from PDB entry 1KTL)^7^ and model **COM**^**∼**^_**Cw7**_ of HLA-Cw7 (peptide taken from PDB entry 3BZF).^16^ Finally, model (viii) **COM_apo_** was generated by removing the nonameric peptide from the model **COM^+^_G_** (Movie S1). The simulated eight models along with the additional data are shown in Table 1. The superscript symbols in the immune complex nomenclature denote models that confer protection of target cells from being killed by NK cells (^+^), models lacking NK cell protection (^−^), and models in which NK cell protection is inconclusive (^∼^). All mutations were built using the *tleap* module of Ambertools 18.^29^

**Table 1:** Models of the HLA-E/peptide/β2m/NKG2A/CD94 Immune Complexes Used in MD Simulation Studies with Denoted Sequences of the Nonameric Peptide, HLA-E Allelic Variant, NK Cell Protection, and Model Number

model	nonameric peptide	peptide sequence	HLA-E allelic variant	NK cell protection	model number
**COM**^**+**^**_G_**	G	VMAPRTLFL	*01:01	**yes(+)**(10,11,13,14)	i
^**allele**^**COM**^**+**^**_G_**	G	VMAPRTLFL	*01:03	**yes(+)**(10,11,13,14)	ii
**COM**^**+**^_**B7**_	B7	VMAPRTVLL	*01:01	**yes(+)**(10,11,13,14)	iii
**COM^**–**^**_**Hsp60sp**_	Hsp60sp	QMRPVSRVL	*01:01	**no(−)**(12,13)	iv
**COM**^**–**^_**B7_R5V**_	B7 R5V	VMAPVTVLL	*01:01	**no(−)**(12)	v
**COM**^**∼**^_**B27**_	B27	VTAPRTLLL	*01:01	**inconclusive(∼)**(10,11,13,15,47)	vi
**COM**^**∼**^_**Cw7**_	Cw7	VMAPRALLL	*01:01	**inconclusive(∼)**(11,13,14,47)	vii
**COM**_**apo**_			*01:01	**N/A**	viii

### Molecular Dynamics (MD) Simulations

Classical molecular dynamics simulations (MD) were performed using the Amber 18 PMEMD software package,^29^ and the AMBER-ff14SB force field (FF) was used for proteins.^30^ Protonation states of ionizable residues were determined using the PDB2PQR web tool under a neutral pH condition of 7.^31^ Carboxylic amino acids were found in their common deprotonated states, whereas histidines were protonated at Nε, Nδ, or both positions. The system was embedded in a 10 Å layer of TIP3P water molecules,^32^ resulting in a box of 126.653 × 134.686 × 123.583 Å^3^, together with 19 Na^+^ counterions, and water molecules counted up to 206 859 atoms. All disulfide bonds were built using the *tleap* module of Ambertools 18,^29^ which was also used to prepare the topologies of the models.

After the initial minimization, the system was gradually heated to 303 K in two sequential steps—0–100 K over 5 ps in the first step and 100–303 K over the next 120 ps in the second step. Positional restraints of 200 and 100 kcal/mol Å^2^ on the heavy atoms were used, respectively. Next, the restraints were removed, and 10 ns of isothermal–isobaric ensemble (*NPT*) function was performed, where pressure control (1 bar) was achieved using a Berendsen barostat.^33^ Productive MD was conducted for the canonical ensemble (*NVT*) using periodic boundary conditions for 1.2 μs for each model, resulting in a total simulation time of ∼10 μs. During the MD simulations, temperature control (303 K) was performed using the Langevin thermostat^34^ with a collision frequency of 1 ps^–1^. The SHAKE algorithm^35^ was used to constrain bonds of hydrogens, and the particle mesh Ewald method^36^ with a cutoff of 10 Å was used to account for long-range electrostatic interactions. An integration time step of 2 fs was set during all MD runs.

VMD^37^ and PyMol^38^ software tools were used for visualization and inspection of trajectories, respectively. MD trajectory analyses, including root-mean-square fluctuations (RMSF), and calculation of the cross-correlation matrices were performed with the *cpptraj* module of Ambertools 18^29^ and with the Gromacs 2016^39^ suite on the stripped trajectories without water and counterions, considering only the equilibrated part of the trajectories (the last 1 μs of the production run, corresponding to the last 10,000 frames, between which three strides were taken, leaving 3334 frames).

H-bonds were determined using the *cpptraj* module of Ambertools 18.^29^ A distance cutoff of 3.0 Å and an angle cutoff of 135° were used as geometric considerations to account for a formed hydrogen bond. Structural populations were settled with a cluster analysis of the trajectories using the *cpptraj* module of Ambertools 18.^29^ Here, a hierarchical agglomerative approach, a distance cutoff of ∼2 Å, and a distance metric of the mass-weighted rmsd of atoms were employed.^40^

### Cross-Correlation Matrices and Correlation Scores

The cross-correlation matrices, based on Pearson’s correlation coefficients (*CCij*), quantify correlated and anti-correlated motions between pair residues along the MD trajectory. *CCij* values can range from −1, indicating completely anti-correlated motion between two residues, to +1, indicating correlated motion; meanwhile, 0 indicates no correlation.

First, the covariance matrices were built from the atom position vectors. To capture only the internal dynamics of the complex, a RMS-fit to a reference structure (an averaged structure from the MD run) was performed, removing the rotational and translational motions as previously described.^41−43^ Next, the cross-correlation matrices (or normalized covariance matrices) were calculated from the covariance matrices with the aid of the *cpptraj* module of Ambertools 18.^29^ In order to make the relationships between individual proteins and domains of the immune complex immediately clear, the correlations for each protein/domain pair were assessed by summing the correlation scores (CSs) between each protein/domain and all others. Next, a correlation density for each area was obtained by summing the CSs of the protein/domain pair, which was then divided by the product of the number of residues belonging to that pair of proteins/domains. This approach eventually led to a simplified variant of the *CCij* matrices.^44,45^

### Binding Free Energy and Interaction Energy Calculations

The binding free energies between the complexes HLA-E/β2m/NKG2A/CD94 and the corresponding nonameric peptide and between NKG2A/CD94 (receptor) and HLA-E/β2m/peptide (ligand) were calculated using the molecular mechanics/generalized born surface area (MM-GBSA) method^46^ and Amber18 code^29^ with pairwise and per-residue decomposition. The value of the igb flag was set to 5, and a salt concentration of 0.1 M was used. MM-GBSA calculations were performed for 100 equally distant frames from each MD trajectory in the production simulation time interval between 600 and 900 ns. The conformational entropic contribution of free energy was not included in the calculations because it was previously suggested that this term does not improve the quality of the results when using MM-GBSA.^46^ The interaction energies between HLA-E/β2m/NKG2A/CD94 and the corresponding nonameric peptide were also calculated using the gmx energy module of the Gromacs2016^39^ software package.

## Results

**Figure 1:**
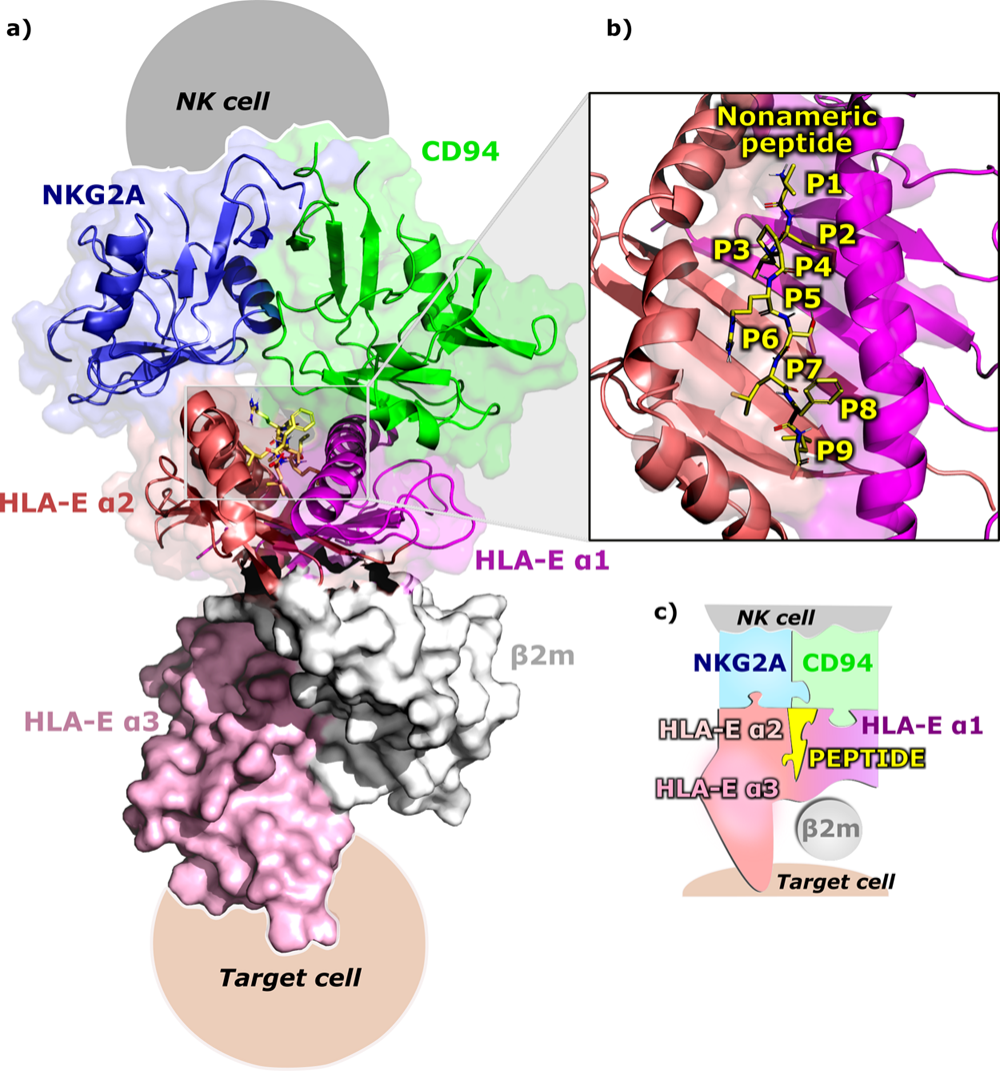
Immune complex of a general model consisting of NKG2A (blue), CD94 (green), HLA-E (α1 and α2 domains), and nonameric peptide G (yellow) depicted in cartoon representation with a transparent surface. HLA-E is further divided into α1 (violet), α2 (salmon), and α3 (pink) subdomains of the HLA-E heavy chain and the beta-2 microglobulin (β2m; gray) light chain, where α3 domain and light chain are portrayed in a solid surface representation. (b) Placement of the nonameric peptide (yellow) between the α1 and α2 domains of HLA-E in the binding pocket is represented by the transparent surface. For clarity, only polar hydrogens are displayed. (c) Schematic representation of the immune complex in the form of differently colored and shaped puzzles.

All models simulated in this study were firmly based on the available crystal structure of the extracellular domains of the human CD94/NKG2A in complex with HLA-E (PDB entry: 3CDG). Our models include all resolved components consisting of NKG2A, CD94, HLA-E, β2m, and nonameric peptide nested between the α1 and α2 domains of the HLA-E (Figure 1).

In our simulations, we evaluated structural features and dynamic behavior of eight models of immune complexes, which are presented in Table 1: (i) base model with peptide from the HLA-G leader sequence (**COM^+^_G_**), (ii) complex of allelic variant *01:03 of HLA-E (^**allele**^**COM^+^_G_**), (iii) complex with peptide derived from HLA-B7 (**COM**^**+**^_**B7**_), (iv) complex with Hsp60 peptide (**COM**^**–**^_**Hsp60sp**_), (v) complex with B7 R5V signal peptide (**COM^–^**_**B7_R5V**_), (vi) complex with B27 peptide (**COM^∼^**_**B27**_), (vii) complex with Cw7 peptide (**COM^∼^**_**Cw7**_), and (viii) complex without peptide (**COM**_**apo**_). Models **COM^+^_G_**, ^**allele**^**COM^+^_G_**, and **COM^+^**_**B7**_ correspond to the NK cell protection scenario; in models **COM^–^**_**Hsp60sp**_ and **COM^–^**_**B7_R5V**_, protection is absent, and in models **COM^∼^**_**B27**_ and **COM^∼^**_**Cw7**_, experiments revealed inconclusive results. The model of allelic variant (^**allele**^**COM^+^_G_**) serves as a replica of a basic model (**COM^+^_G_**), whereas the model without peptide (**COM**_**apo**_) represents a negative control (Movie S1).

The nonameric peptides selected for models of investigated immune complexes bind to HLA-E with different affinities,^7,10−13^ where peptide G, present in complexes **COM^+^_G_** and ^**allele**^**COM^+^_G_**, possesses the highest affinity for the NKG2A/CD94 receptor when incorporated with HLA-E and provides protection against the cytolytic activity of NK cells.^11,14^ The peptide B7, present in **COM^+^**_**B7**_ model, also provides protection against cytolysis by NK cells.^12^ Meanwhile, in cases of Hsp60sp (**COM^–^**_**Hsp60sp**_) and the mutated form of B7 (**COM^–^**_**B7_R5V**_), such protection is missing (Table 1).^12,13^ The NK cell protection ability of models **COM^∼^**_**B27**_(10,11,13,15,47) and **COM^∼^**_**Cw7**_(11,13,14,47) is inconclusive from the current experimental results. In the following subsections, the subtleties between the studied models and their corresponding peptides are addressed from a geometric and energetic point of view.

### HLA-E α2 Potentially Facilitates the Interaction between the Nonameric Peptide and NKG2A Protein

**Figure 2:**
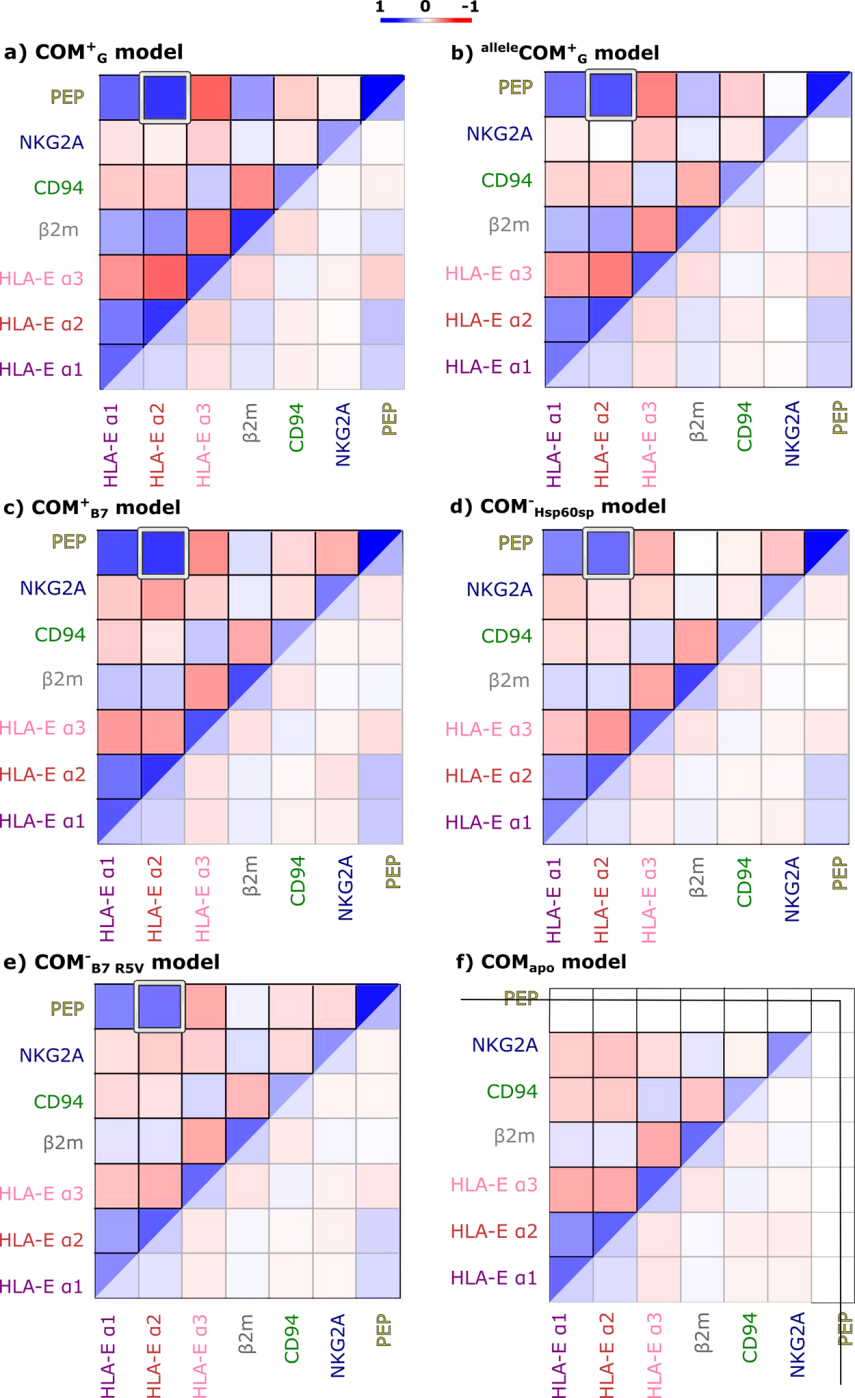
Simplified cross-correlation matrices for the simulated models (a) **COM^+^_G_**, (b) ^**allele**^**COM^+^_G_**, and (c) **COM^+^**_**B7**_ that all provide NK cell protection, models (d) **COM^–^**_**Hsp60sp**_ and (e) **COM^–^**_**B7_R5V**_ with absent NK cell protection, and model (f) **COM**_**apo**_. More pronounced correlation and anti-correlation patterns were observed in the complex **COM^+^_G_** model as depicted by more intense colors.

Consistent with previous reports of the “lock and key”-like engagement between the ligand and the receptor,^24^ several microsecond-long MD simulations of all eight investigated models did not detect any major conformational differences associated with either activation or inhibition of NK cells via this complex. In order to explore the internal dynamics, we further constructed and analyzed the cross-correlation matrices (*CCij*) and derived their simplified versions for a clearer presentation (see the Methods section). Results for the simulated models **COM^+^_G_**, ^**allele**^**COM^+^_G_,COM^+^**_**B7**_, **COM^–^**_**Hsp60sp**_, and **COM^–^**_**B7_R5V**_ with a conclusive effect on the NK cells and **COM**_**apo**_ model are presented in Figure 2 as cross-correlation matrices; meanwhile, the remaining data can be accessed in Figures S1 and S2.

Analysis of the simplified cross-correlation matrices of all subjected models pointed toward similar patterns. This observation suggests that protein–protein correlations are generally preserved regardless of the productive or non-productive receptor–ligand recognition, leading to NK cell inhibition or lack thereof. Furthermore, the cross-correlation map obtained for the **COM**_**apo**_ model shows that the bound nonameric peptide does not lead to any major conformational changes in the studied immune complexes. However, upon a detailed examination of the cross-correlation matrices, the correlation and anti-correlation patterns were more pronounced for the complexes **COM^+^_G_**, ^**allele**^**COM^+^_G_**, and **COM^+^**_**B7**_, mediating successful protection against the NK cells (Figures 2 and S1). This observation could be linked to the ability of the nonameric peptides incorporated into these complexes to promote a conformation responsible for mediating successful protection. Contrarily, in other investigated complexes (models **COM^∼^**_**B27**_, **COM^∼^**_**Cw7**_, **COM^–^**_**Hsp60sp**_, and **COM^–^**_**B7_R5V**_), the cross-correlation matrices display a less-pronounced averaged (anti)correlation relations.

The cross-correlation matrices also revealed that nonameric peptides G and B7 present in models **COM^+^_G_**, ^**allele**^**COM^+^_G_**, and **COM^+^**_**B7**_ appear to have a stronger correlation with HLA-E α2 compared to peptides present in the investigated **COM^–^**_**Hsp60sp**_ and **COM^–^**_**B7_R5V**_ models (Figures 2 and S1). This suggests that close cooperation between the nonameric peptide and HLA-E α2 region on the target cell is necessary for successful recognition by the NK receptor NKG2A/CD94 and protection against NK killing.

According to the rmsd trajectory analysis, the peptides in **COM^+^_G_**, ^**allele**^**COM^+^_G_**, and **COM^+^**_**B7**_ models mediating NK cell protection generally have lower rmsd values (Figure S3). The average rmsd values obtained for complexes **COM^+^_G_**, ^**allele**^**COM^+^_G_**, and **COM^+^**_**B7**_ were 1.8 ± 0.3, 1.9 ± 0.4, and 1.8 ± 0.3 Å, respectively. On the other hand, in models **COM^–^**_**Hsp60sp**_ and **COM^–^**_**B7_R5V**_, the average rmsd values 2.8 ± 0.3 and 2.2 ± 0.3 Å, respectively, were calculated (Figure S3). It is important to mention that the observed higher rmsd values could in part be also related with the changes introduced into the initial peptide sequence.

**Figure 3:**
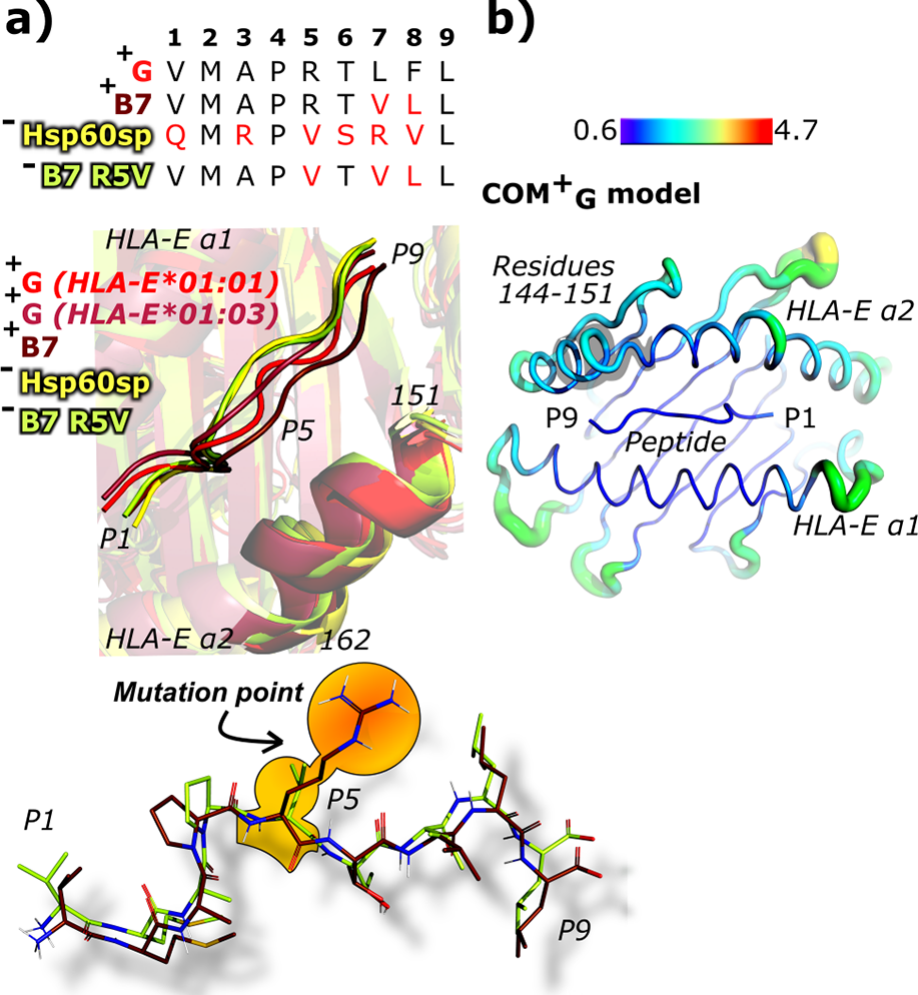
Comparison of the representative conformations of the nonameric peptides in the binding pockets of different model complexes with a conclusive NK cell protection status. (a) Sequence alignment of the peptides G, B7, Hsp60sp, and B7 R5V. Residues that differ from the peptide G sequence are marked in red. Alignment of cartoon representations of nonameric peptides in the models **COM^+^_G_** (red), **^allelic^COM^+^_G_** (pink), **COM^+^**_**B7**_ (brown), **COM^–^**_**Hsp60sp**_ (yellow), and **COM^–^**_**B7_R5V**_ (green) shows apparent differences in the P5 position. The area of the HLA-E α2 domain from the residues 151–162 is also highlighted. Alignment of nonameric peptides B7 and B7 R5V with a highlighted mutation point. (b) Flexibility of **COM^+^_G_** HLA-E α1 and α2 domains and nonameric peptide depicted as the B-factor representation with a shaded area of the HLA-E 2α between the residues 144 and 151.

After visual inspection of the most representative clusters extracted from the MD simulation trajectories for each simulated model, we noticed evident positional differences of the amino acid at the P5 position among the simulated nonameric peptides G, B7, B27, Cw7, Hsp60sp, and B7 R5V relative to the HLA-E α2 domain (Figures 3a, S4). We further quantitatively confirmed this observation by measuring the distances between the Cα atom of the P5 nonameric peptide and the center of the alpha helix between residues 151 and 162 of the HLA-E α2 domain in the most representative clusters. The distances for the models containing the peptides G, B7, B27, and Cw7 were around 9 Å, whereas in models with the peptides Hsp60sp and B7 R5V, they were 12 Å and 11 Å, respectively. This observation is most likely associated with the absence of the Glu152^HLA-E^–Arg^P5^ salt bridge in the models **COM^–^**_**Hsp60sp**_ and **COM^–^**_**B7_R5V**_.

Visualization and RMSF analysis of local flexibility within our eight simulated immune complexes revealed that the α1 domain of the HLA-E heavy chain is more rigid in the regions closer to the nonameric peptide compared to its α2 domain, which is somewhat more flexible, especially at the C-terminal side of the peptide, between the residues 144 and 151 (Figures 3b, S5 and S6). Furthermore, we also observed at least comparable flexibility of the nonameric peptide, although the HLA-E α2 domain may even be more flexible than the bound peptides (Figures S6 and S7). The most flexible part of the peptide is mostly located at its C-terminal side, while the N-terminus is more rigid. This later observation might be connected with the higher flexibility of the HLA-E α2 domain that is in close contact with the C-terminal side of the peptide.

No specific contacts between the peptide and the NKG2A protein were observed in our simulations, consistent with what has been reported in the literature.^24^ However, observed changes in the HLA-E α2 domain flexibility could play a role in the inhibitory signal transduction mediated via NKG2A/CD94 on the NK cells.

### Hydrogen Bonding Network Guides the Receptor–Ligand Molecular Recognition

After a close-up inspection, we noticed notable differences in the intermolecular interactions between the nonameric peptide, HLA-E, CD94, and NKG2A proteins. To begin with, the Glu152^HLA-E^–Arg^P5^ salt bridge was present in simulated models with G, B7, B27, and Cw7 bound peptides (**COM^+^_G_**, **^allele^COM^+^_G_**, **COM^+^**_**B7**_, **COM^∼^**_**B27**_, and **COM^∼^**_**Cw7**_ models), while in the models with Hsp60sp or B7 R5V (**COM^–^**_**Hsp60sp**_, **COM^–^**_**B7_R5V**_) with absent NK cell protection, this interaction was absent, which is in accordance with the literature data.^28^

**Figure 4:**
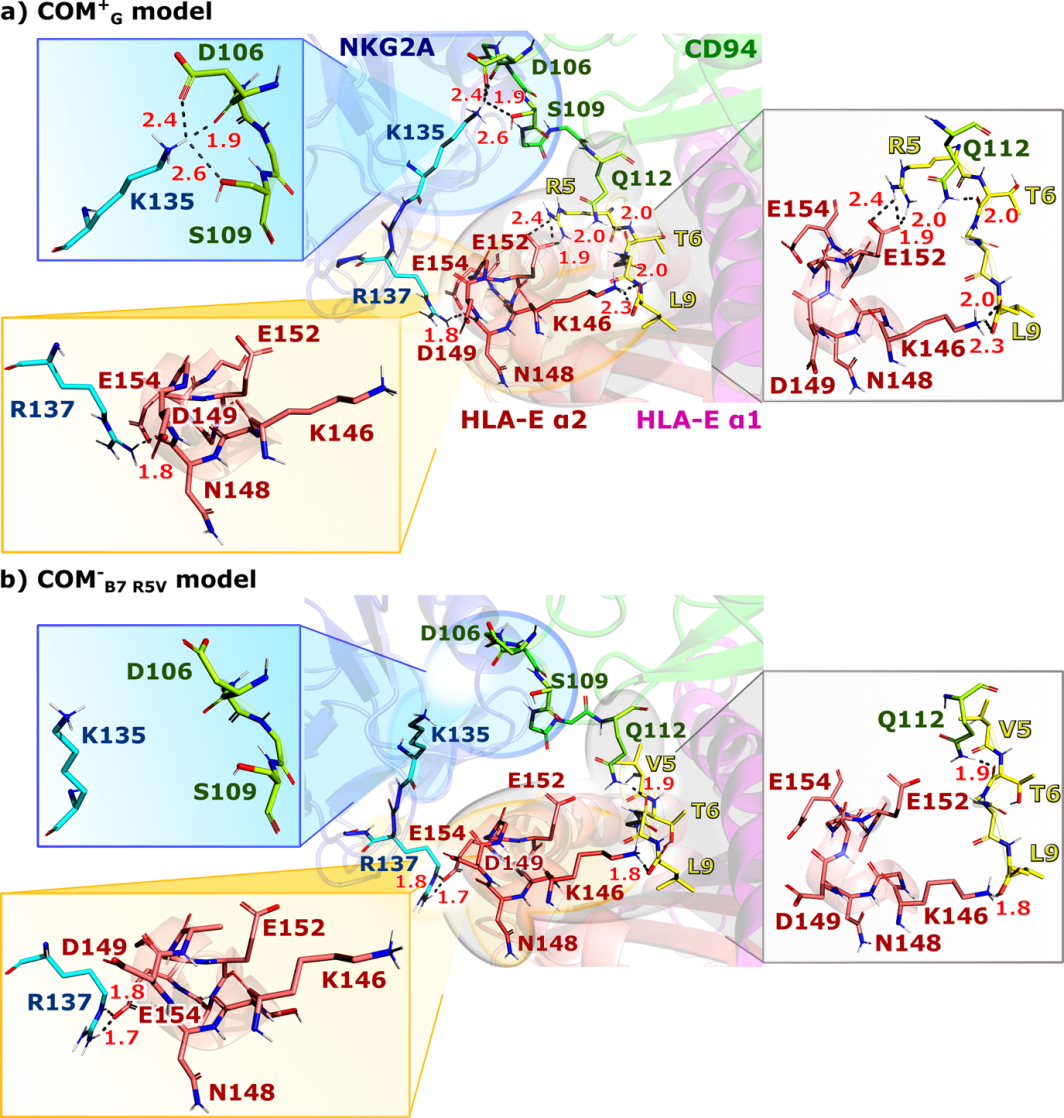
Comparison of the observed molecular recognition network in the representative models with (a) present (model **COM^+^_G_**) or (b) absent (model **COM^–^**_**B7_R5V**_) NK cell protection outcomes. Key interactions derived from the most representative clusters of the simulations are magnified in separate boxes for each model; the blue box depicts interactions between the NKG2A and CD94, the orange box shows positioning of Arg137^NKG2A^ relative to the HLA-E α2 domain, and the white box represents key interactions between the nonameric peptide, HLA-E α2 domain, and CD94 protein. For clarity, only polar hydrogens are shown.

Additionally, we observed an H-bond between Gln112^CD94^ and the backbone oxygen of the Thr^P6^ (Ala^P6^ for **COM^∼^**_**Cw7**_) in all models except **COM^–^**_**Hsp60sp**_, where instead, a quite analogous H-bond between Gln112^CD94^ and the backbone nitrogen of the Val^P8^ is sparsely present. The H-bond interaction between Lys146^HLA-E^ and the backbone oxygen of the highly conserved Leu^P9^ residue located at the C-terminus of the nonameric peptide was present in the most representative clusters extracted from the MD simulation trajectory of all peptide-enclosing models except **COM^–^**_**Hsp60sp**_ and **COM^∼^**_**B27**_, where the interaction was missing. Furthermore, the Lys135^NKG2A^–Asp106^CD94^ salt bridge and Lys135^NKG2A^–Ser109^CD94^ H-bond were present in models **COM^+^_G_**, **^allele^COM^+^_G_**, and **COM^+^**_**B7**_, enabling NK cell protection, and were not noticeable in the most representative clusters of all other models studied by MD (Figures 4, S8–S26, and Table S1). It should also be noted that no particular hydrophobic interactions important for peptide binding were observed during the analysis of MD trajectories.

Local interaction analysis of our simulations revealed that the Glu152^HLA-E^–Arg^P5^ salt bridge and Gln112^CD94^–Thr^P6^ (Ala^P6^ for **COM^∼^_Cw7_**), Lys135^NKG2A^–Asp106^CD94^, and Lys135^NKG2A^–Ser109^CD94^ H-bond interactions, as presented in Figure 4, appear to be associated with the ability of successful recognition of the HLA-E/β2m/peptide ligand by the inhibitory NK cell receptor NKG2A/CD94. Thus, this network of molecular recognition elements could play an important role in the intricate machinery of cell protection against the NK cell-induced cytotoxicity.

### Binding Free Energy and Interaction Energy Analysis Pinpoints Important Protein–Protein Contacts

Binding free energies (Δ*G*_b_) were calculated between the nonameric peptide and the HLA-E/β2m/NKG2A/CD94 complex and between the NKG2A/CD94 (receptor) and HLA-E/β2m/peptide (ligand) entities using the MM-GBSA method.^46^ With the pairwise decomposition of ΔG_b_, we identify at the atomic level the energetically most important interactions, and with the per-residue decomposition, we calculated the energy contribution of each single residue by summing its interactions over all residues in the systems. Residues with the highest per-residue contribution can also be referred to as hotspots.^48^

The nonameric peptide complex energies correlate well with the reported NK cell protection ability. The studied models **COM^+^_G_**, **^allele^COM^+^_G_**, and **COM^+^**_**B7**_ generally have more favorable Δ*G*_b_ compared to models **COM^–^**_**Hsp60sp**_ and **COM**_**^–^B7_R5V**_ (Tables 2, S2 and 3, and Figure S27). The same observation was made when we performed the interaction energy calculations using the gmx energy module of Gromacs2016^39^ (Table S4). Here, the value of the **COM^∼^**_**Cw7**_ interaction energy is located between the values determined for the **COM^+^**_**B7**_ and **COM^–^**_**Hsp60sp**_ models; meanwhile, the inconclusive model **COM^∼^**_**B27**_ is positioned between the **COM^–^**_**Hsp60sp**_ and **COM^–^**_**B7_R5V**_ models in terms of interaction energy. The latter also correlates with our findings from the interaction analysis of the **COM^∼^_Cw7_** model geometric properties, showing that the **COM^∼^**_**Cw7**_ system exhibits more similar features to the models with reported successful ligand–receptor recognition and NK cell protection, whereas model **COM^∼^**_**B27**_ traits are more similar to the models **COM^–^**_**Hsp60sp**_ and **COM^–^**_**B7_R5V**_.

**Table 2:** Binding Free Energies (ΔG_b_) between Nonameric Peptide and Remaining HLA-E/β2m/NKG2A/CD94 Calculated by the MM-GBSA Method and Its (a) Pairwise and (b) Per-Residue Decomposition (in kcal/mol) for the Selected **COM^+^_G_** and **COM^–^**_**B7_R5V**_ Models and **COM^+^_G_** Model^a^

(a)							
**COM^+^_G_** Δ*G*_b_ –119.1 ± 7.4 [kcal/mol]				**COM^–^**_**B7_R5V**_ Δ*G*_b_ –90.7 ± 7.5 [kcal/mol]			
res 1	res 2	Δ*G*_b_ total	type	res 1	res 2	Δ*G*_b_ total	type
*CD94*				*CD94*			
**Gln112**	**Thr**^**P6**^	**–3.9±0.0**	**H-bond**	**Gln112**	**Thr**^**P6**^	**–3.6±0.5**	**H-bond**
Gln112	Phe^P8^	–3.1 ± 0.5	HI^b^	Gln112	Leu^P8^	–1.7 ± 0.4	HI
Asn160	Phe^P8^	–2.1 ± 0.9	cation-π	Asn160	Leu^P8^	–0.8 ± 0.2	HI
Gln112	Arg^P5^	–1.4 ± 1.0	cation-π	Gln112	Val^P5^	–1.1 ± 0.4	HI
Asn158	Phe^P8^	–1.3 ± 0.4	cation-π	Gln113	Val^P5^	–1.3 ± 0.4	HI
*HLA-E*				*HLA-E*			
Glu63	Val^P1^	–23.9 ± 4.5	H-bond	Glu63	Val^P1^	–23.9 ± 3.5	H-bond
**Glu152**	**Arg**^**P5**^	**–19.2±2.0**	**H-bond**	**Lys146**	**Leu**^**P9**^	**–13.7±5.2**	**H-bond**
**Lys146**	**Leu**^**P9**^	**–14.0±4.6**	**H-bond**	Ser143	Leu^P9^	–6.9 ± 1.5	H-bond
Gln156	Arg^P5^	–9.6 ± 0.9	H-bond	Tyr171	Val^P1^	–5.6 ± 1.1	H-bond
Ser143	Leu^P9^	–6.9 ± 1.2	H-bond	Tyr7	Val^P1^	–5.1 ± 1.6	H-bond

(b)	
**COM^+^_G_** Δ*G*_b_ –119.1 ± 7.4 [kcal/mol]	
residue	Δ*G*_b_ total
**Met**^**P2**^	**–11.6±1.4**
**Phe**^**P8**^	**–9.6±1.2**
**Leu**^**P9**^	**–8.9±2.6**
Leu^P7^	–6.4 ± 0.9
Arg^P5^	–5.8 ± 1.8
Thr^P6^	–4.0 ± 1.4
Ala^P3^	–2.4 ± 0.5
Pro^P4^	–1.4 ± 0.3
Val^P1^	1.1 ± 2.1

a The table lists top five interactions between the nonameric peptide–CD94 and nonameric peptide–HLA-E pair of residues together with the type of interaction.

b HI—hydrophobic interaction.

Obtained results of the pairwise energy decomposition revealed that P1, P5, P6, P8, and P9 residues of the nonameric peptide interactions seem to contribute the most to the binding free energy ΔG_b_ between the peptide and the remaining HLA-E/β2m/NKG2A/CD94 immune complex (Tables 2 and S2 and S5). This is in line with the interactions of the immune complex HLA-E/β2m/peptide G/CD94/NKG2A reported in the literature.^24^ Interestingly, the H-bond interactions, namely, Glu152^HLA-E^–P5, Lys146^HLA-E^–Leu^P9^, and Gln112^CD94^–P6, are among those that have the largest contributions to the Δ*G*_b_ of the nonameric peptide, further reinforcing the observed correlations between the geometric and energetic traits of the studied system.

On the other hand, the per-residue free energy decomposition pinpoints the P2, P8, and P9 residues of the nonameric peptide to generally contribute most to the ΔG_b_ between HLA-E/β2m/NKG2A/CD94 and the corresponding nonameric peptide (Tables 2, S3 and Figure S27) among all nine peptide residues. This is consistent with previous reports that P2 and P9 are considered the dominant peptide anchor positions^28^ and that P8 and P6 (along with P5) residues form the majority of contacts of the peptide with the NKG2A/CD94 receptor.^28^

**Figure 5:**
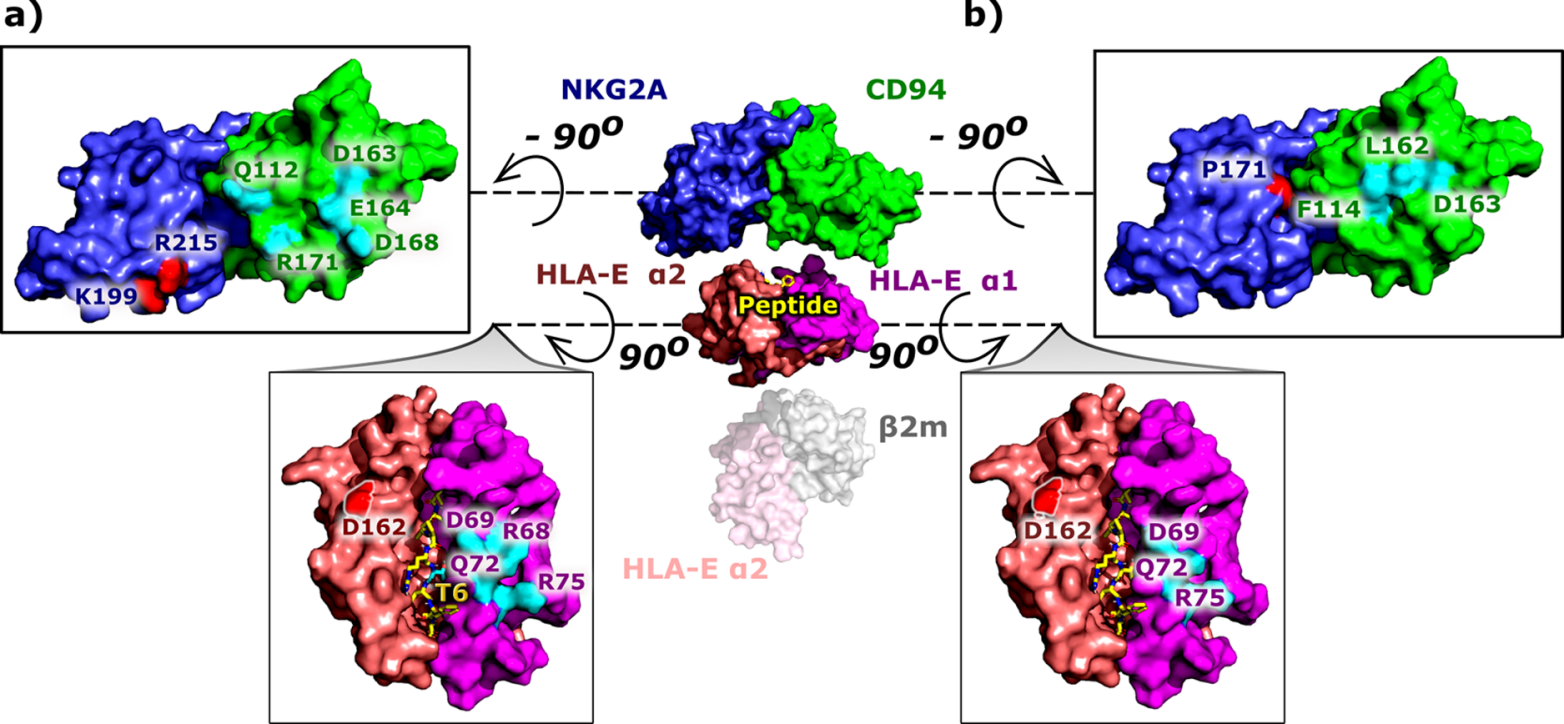
Receptor–ligand interface with key contacts common for all simulated models. Residues with the greatest contribution to the binding free energy between the ligand—HLA-E/β2m/peptide (shown in magenta, salmon, pink, gray, and yellow, respectively)—and the receptor—NKG2A/CD94 (in blue and green, respectively)—with (a) pairwise and (b) per-residue decomposition, shown in surface and licorice representation (peptide). Residues are colored in red and cyan for HLA-E α1-NKG2A and HLA-E α2-CD94 interacting pairs, respectively. The number of colored residues on the receptor surface indicates the great importance of the CD94 protein in receptor–ligand interactions.

Next, pairwise free energy decomposition between the receptor–ligand entities identifies seven consistent interactions with the greatest contribution to the Δ*G*_b_, in the models **COM^+^_G_**, **^allele^COM^+^_G_**, and **COM^+^**_**B7**_, exhibiting successful protection against the NK cells, which are only partially present in **COM^–^**_**Hsp60sp**_ and **COM^–^**_**B7_R5V**_ models with absent recognition. These interactive pairs are Asp69^HLA-E^–Arg171^CD94^, Arg75^HLA-E^–Asp163^CD94^, Asp162^HLA-E^–Lys199^NKG2A^, Gln72^HLA-E^–Glu164^CD94^, Arg68^HLA-E^–Asp168^CD94^, Asp162^HLA-E^–Arg215^NKG2A^, and Thr^P6^–Gln112^CD94^ (Figures 5 and S28 and Tables S6 and S7).

Lastly, the per-residue free energy decomposition between the receptor–ligand entities unraveled consistent occurrence of eight residues contributing most to their productive binding in **COM^+^_G_**, **^allele^COM^+^_G_**, and **COM^+^**_**B7**_ models. These residues were Asp162^HLA-E^, Asp69^HLA-E^, Gln72^HLA-E^, Arg75^HLA-E^, Phe114^CD94^, Asp163^CD94^, Leu162^CD94^, and Pro171^NKG2A^. All eight residues are also present in the model **COM^–^**_**Hsp60sp**_, while two of them are missing in the model **COM^–^**_**B7_R5V**_. Energy decomposition also showed that the residues at P6 and P8 positions of the peptide are among the top 10 ligand residues with the greatest contribution to the Δ*G*_b_ in all models except **COM^+^**_**B7**_ and **COM^–^**_**Hsp60sp**_ (Figures 5 and S28 and Table S8).

Based on the consistency of contributions of the aforementioned eight residues to the Δ*G*_b_ obtained by the per-residue free energy decomposition analysis of all models, they could be considered as potential hotspots due to their large contribution to the binding free energy.^48^ It was gratifying to observe that the ligand–receptor recognition of binding affinity was also previously attributed to all but one of the proposed hotspots. Indeed, mutations of Asp162^HLA-E^, Arg75^HLA-E^,^27^ Phe114^CD94^, and Leu162^CD94^^26^ to Ala were found to abolish successful molecular recognition between the HLA-E/2m/peptide and NKG2A/CD94 binding partners. Meanwhile, mutations of Gln72^HLA-E^, Asp69^HLA-E^,^26,27^ and Asp163^CD94^^26^ to alanine resulted in impaired binding.

The free energy calculations reproduced several experimentally discovered hotspots and showed that the CD94 protein forms the majority of the energetically important interactions with the HLA-E/β2m/peptide ligand, primarily with the HLA-E α1, thus being the core of molecular recognition and successful complex formation (Figure 5).

## Discussion

In our study, we generated eight complexes of the HLA-E/β2m/NKG2A/CD94 system with nonameric peptides in an attempt to elucidate the mechanistic details guiding the receptor (NKG2A/CD94) molecular recognition of the ligand (HLA-E/β2m/peptide) at the atomistic level.

**Figure 6:**
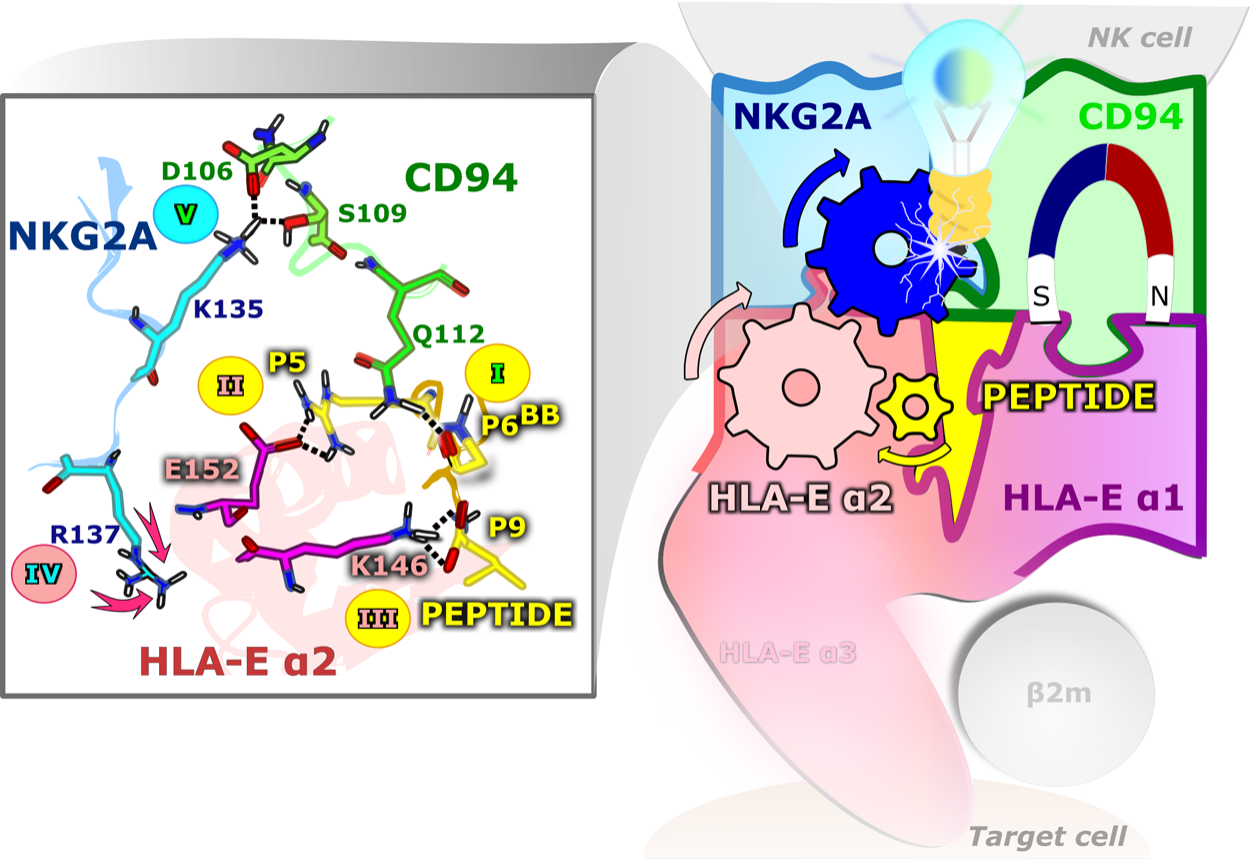
Schematic representation of the possible NK cell inhibition via the proposed hydrogen bond network in the immune complex starting from the nonameric peptide via the HLA-E α2 domain to the receptor NKG2A protein as revealed by MD simulations. On the left panel, the numbers show the possible sequence of events in the peptide/HLA-E α2/NKG2A/CD94 complex leading to successful NK cell protection. The receptor’s CD94 and the ligand HLA-E α1 protein domain form the strongest interactions in the system and are thus responsible for the core of receptor–ligand recognition.

Multi-microsecond-long all-atom MD simulations based on the available experimental (NKG2A/CD94)–(HLA-E/β2m/peptide) complex crystal structures showed that no major conformational changes occur during the molecular recognition event taking place between the receptor and ligand. Furthermore, no specific interactions between the NKG2A protein and nonameric peptide residues could be detected. The analysis of the MD-generated conformations of all simulated systems further pinpointed that the CD94 receptor protein forms most of the energetically important contacts with the ligand, which underlines its core anchoring role in the receptor–ligand recognition. Given the observed HLA E α2-flexibility in the region near the C-terminus of the peptide, it is possible that the interaction between the nonameric peptide and NKG2A protein occurs via the α2-domain of HLA-E (Figure 6).

Our simulation study confirmed many previously experimentally reported contacts and interactions between CD94/NKG2A and HLA-E/β2m/peptide thought to be important for the receptor–ligand recognition including the crucial role of Arg^P5^,^12,24^ which forms an H-bond with Glu152^HLA-E^, and the importance of Gln112^CD94^,^24,26^ which forms an H-bond with the peptide’s P6 residue (Movie S1 and Figure 4). Additionally, we corroborated several hotspots at the receptor–ligand interface (Figure 5) that could serve as starting points in the development of potential inhibitors of protein–protein interactions, leading to the formation of this complex. The majority of them have been previously reported by experimental alanine scanning.^24,26,27^ Moreover, we detected previously unreported H-bonds between CD94 and NKG2A proteins, namely, Asp106^CD94^–Lys135^NKG2A^ and Ser109^CD94^–Lys135^NKG2A^, which are more pronounced in the complexes **COM^+^_G_**, **^allele^COM^+^_G_**, and **COM^+^**_**B7**_ with peptides that provide successful ligand–receptor recognition and protection from being killed by NK cells.

Interestingly, there also appeared to be a slight difference in the positioning of Lys135^NKG2A^ in the simulated immune complexes **COM^+^_G_**, **^allele^COM^+^_G_**, and **COM^+^**_**B7**_, providing protection from NK cell killing. In these systems, Lys135^NKG2A^ was naturally positioned closer to its CD94-binding partners; however, the positioning of Asp106^CD94^ and Ser109^CD94^ did not follow any obvious pattern that would allow us to distinguish if a particular model enables protection from NK cell killing. The plausible reason for this can be the H-bond formed between Gln112^CD94^ and the backbone oxygen of either the P6 or P8 residue of the nonameric peptide in all models, which might be sufficient for the approximately correct positioning of Asp106^CD94^ and Ser109^CD94^ residues. Indeed, mutation of the Gln112^CD94^ > Ala has been reported to abolish binding of CD94/NKG2A to the HLA-E/β2m/peptide,^26^ providing evidence for its core involvement in the recognition event. However, the explanation for a more specific positioning of Lys135^NKG2A^ remains unclear.

Although the simulations do not provide the time series of the events, we hypothesized an intricate key network for the Asp106^CD94^–Lys135^NKG2A^ and Ser109^CD94^–Lys135^NKG2A^ interactions to occur (Figure 6) comprising the following key events. First, (i) a Gln112^CD94^-P6 contact needs to be established to properly position the Asp106^CD94^ and Ser109^CD94^ residues. Then, (ii) Glu152^HLA-E^–Arg^P5^ H-bond formation drives the placement of the HLA-E α2 domain and thus enables the interaction between the peptide and NKG2A protein. It seems likely that (iii) the Leu^P9^–Lys146^HLA-E^ interaction also influences the HLA-E α2 positioning. Furthermore, (iv) HLA-E α2 affects the position of the Arg137^NKG2A^, which is located in its vicinity (Figure S29), which subsequently contacts Lys135^NKG2A^, ultimately allowing (v) the formation of key interactions between the NKG2A and CD94 proteins (Asp106^CD94^–Lys135^NKG2A^ and Ser109^CD94^–Lys135^NKG2A^). Indeed, Arg137^NKG2A^ frequently forms interactions with the α2-residues Asn148^HLA-E^ and Asp149^HLA-E^ in the **COM^+^_G_**, **^allele^COM^+^_G_**, and **COM^+^**_**B7**_ models. Energetic analysis further corroborates this proposed network and shows the importance of Gln112^CD94^–P6, Glu152^HLA-E^–Arg^P5^, and Leu^P9^–Lys146^HLA-E^ interactions.

Our computational studies also explored two models, **COM^∼^**_**B27**_ and **COM^∼^**_**Cw7**_, containing peptides, for which inconclusive information could be found in the literature regarding their ability to successfully facilitate receptor–ligand recognition and subsequent NK cell protection. Our simulations showed that the **COM^∼^**_**B27**_ model containing peptide B27 behaved similarly to the **COM^–^**_**Hsp60sp**_ and **COM^–^**_**B7_R5V**_ models without NK cell protection, while the **COM**^**–**^_**Cw7**_ model containing peptide Cw7 bears more resemblance to **COM^+^_G_**, **^allele^COM^+^_G_**, and **COM^+^**_**B7**_ models providing the NK cell protection. The latter is consistent with previous reports that an inefficient receptor–ligand interaction may eventually reach a threshold sufficient to counteract the NK cell activation.^47^

It should be mentioned that with the exception of the **COM**^**+**^_**G**_ model, where **^allele^COM^+^_G_** can be considered as its replica, a single replica per system was run. Nevertheless, in eight long MD simulations performed here, three models (**COM^+^_G_**, **^allele^COM^+^_G_**, and **COM^+^**_**B7**_) acted as positive controls and other two of them (**COM^–^**_**Hsp60sp**_ and **COM^–^**_**B7_R5V**_) served as negative controls. The obtained good correlation of the observed behavior of all these systems with the experimental data suggests that such a setup of molecular systems can at least in part serve as an alternative to the replica approach.

In summary, our multi-microsecond-long molecular dynamics (MD) simulations comprehensively contribute to the understanding of interactions and their complex interrelations that are critical for a successful ligand (HLA-E/β2m/peptide)–receptor (NKG2A/CD94) recognition at the atomistic level in the fundamental immune process event of the natural killer (NK) cell action. The knowledge obtained here could form the basis for the targeted design of new biochemical and structural studies that may pave the way toward unraveling the complex mechanisms that are part of the innate immune system. Moreover, understanding this fundamental mechanism of NK cell protection may further enable rational structure-based design of interventions, for example, in the form of different ligands and small molecules that would affect HLA-E/β2m/peptide and CD94/NKG2A interactions, which are overexpressed under some pathological conditions and in senescent cell accumulation.

## Supporting Information Available

Supporting figures, tables, and structure and simulation parameter files (PDF)
HLA-E/β2m/peptide/NKG2A/CD94 immune complex (MP4)
Structure files (ZIP)

## Notes

All molecular simulations, analysis, and visualization were performed with widely used programs available freely for academic institution—Gromacs 2016, Amber and AmberTools 18, VMD 1.9.3, and pyMol 2.0. The starting structures were obtained from the Protein data Bank public structure database. All procedures and workflows are described in the Methods section. Structure and parameter files used in simulations are provided in the Supporting Information.
