# Supplementary material for: All-Atom Simulations Reveal a Key Interaction Network in the HLA-E/NKG2A/CD94 Immune Complex Fine-Tuned by the Nonameric Peptide
Source: J Chem Inf Model. 2021 Jul 1;61(7):3593–603. doi: 10.1021/acs.jcim.1c00414 (PMC8389527; doi:10.1021/acs.jcim.1c00414)
Supplement: Supplementary file 1 — ci1c00414_si_001.pdf [file ci1c00414_si_001.pdf]

Supporting Information

All-atom Simulations Reveal a Key Interaction Network in the HLA-E/NKG2A/CD94 Immune Complex Fine-tuned by the Nonameric Peptide

Eva Prašnikar[†, ‡], Andrej Perdih[†, &], Jure Borišek[†,*]

[†]National Institute of Chemistry, Hajdrihova 19, 1000, Ljubljana, Slovenia

[&]Faculty of Pharmacy, University of Ljubljana, Aškerčeva 7, 1000 Ljubljana Slovenia

[‡]Graduate School of Biomedicine, Faculty of Medicine, University of Ljubljana, Vrazov trg 2, 1000 Ljubljana, Slovenia

Corresponding author*:

E-mail: jure.borisek@ki.si

Table of contents

Supporting Figures ... *3*
Figure S1. ... 3
Figure S2. ... 4
Figure S3 ... 5
Figure S4. ... 6
Figure S5 ... 7
Figure S6. ... 8
Figure S7. ... 9
Figure S8. ... 10
Figure S9. ... 11
Figure S10. ... 12
Figure S11. ... 13
Figure S12. ... 14
Figure S13 ... 15
Figure S14 ... 16
Figure S15 ... 17
Figure S16 ... 18
Figure S17. ... 19
Figure S18 ... 20
Figure S19 ... 21
Figure S20 ... 22
Figure S21 ... 23
Figure S22 ... 24
Figure S23 ... 25
Figure S24 ... 26
Figure S25. ... 27
Figure S26. ... 28
Figure S27. ... 29
Figure S28 ... 30
Figure S29. ... 31

Supporting Tables ... *32*
Table S1. ... 32
Table S2. ... 33
Table S3. ... 34
Table S4. ... 35
Table S5. ... 36
Table S6. ... 37
Table S7. ... 38
Table S8. ... 39

Supporting Movies ... *40*
Movie S1 ... 40

Supporting Figures

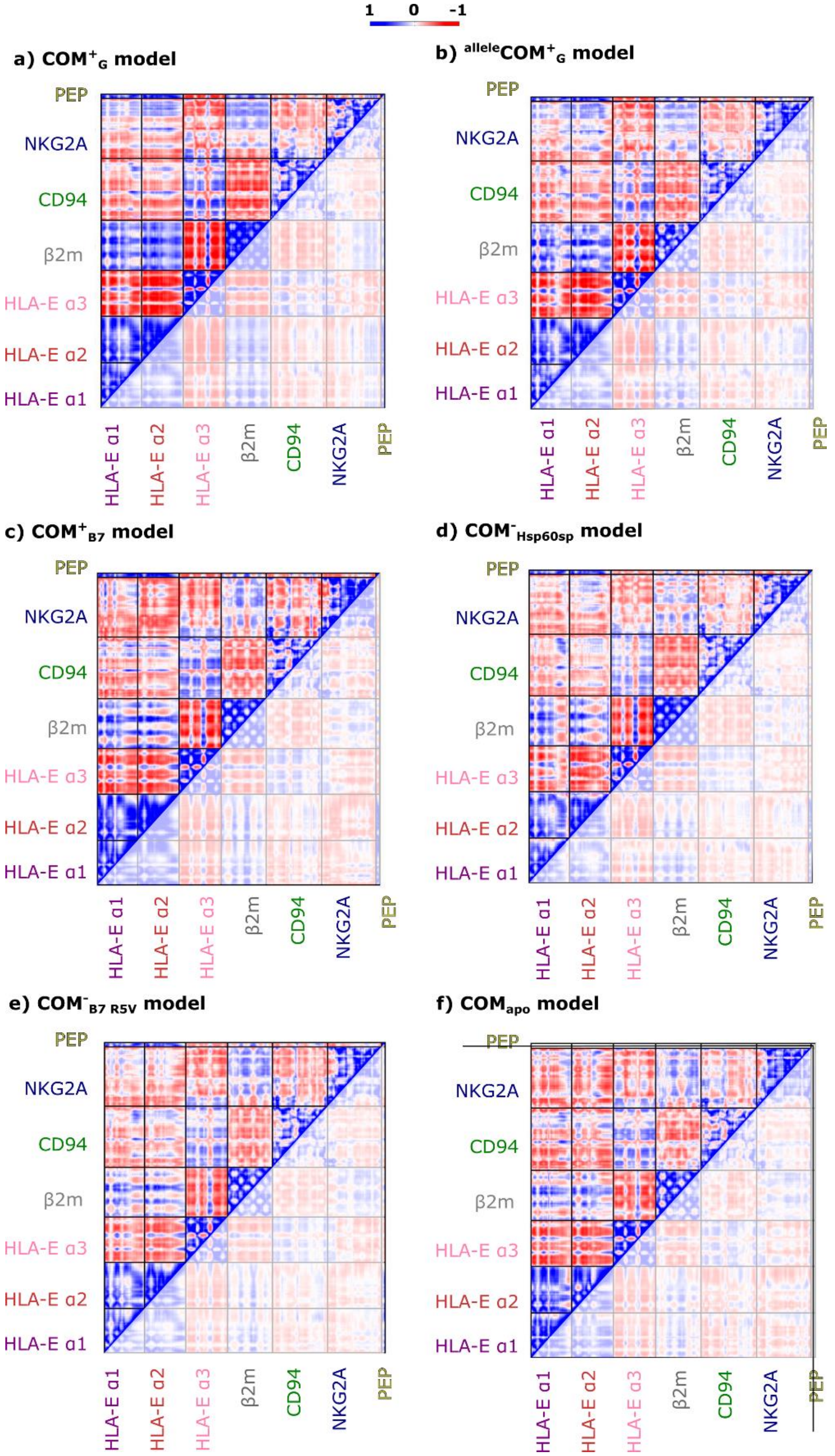

Figure S1. Cross-correlation matrices for models a) $\mathbf{COM^{+}_{G}}$, b) $\mathbf{^{allele}COM^{+}_{G}}$ and c) $\mathbf{COM^{+}_{B7}}$ that provide NK cell protection, models d) $\mathbf{COM^{-}_{Hsp60sp}}$ and e) $\mathbf{COM^{-}_{B7\_R5V}}$ with absent NK cell protection, and model f) $\mathbf{COM_{apo}}$.

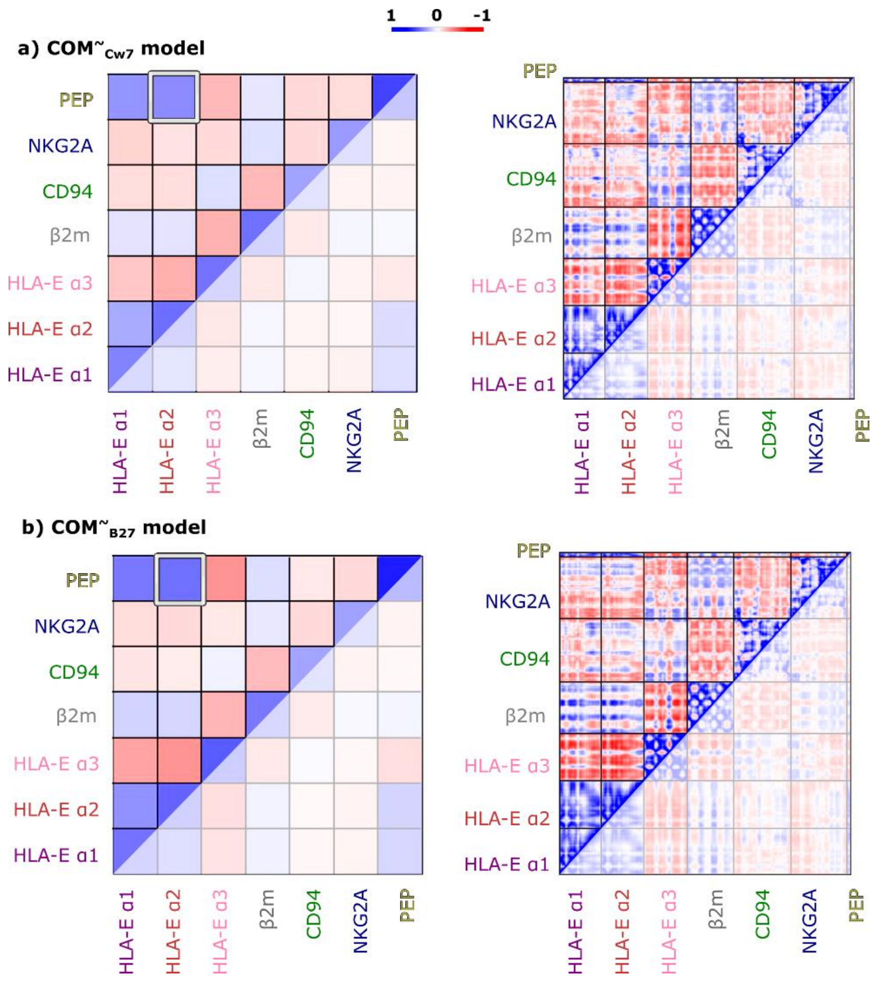

Figure S2. Cross-correlation matrices for models a) **COM˜$_{Cw7}$** and b) **COM˜$_{B27}$** with inconclusive NK cell protection.

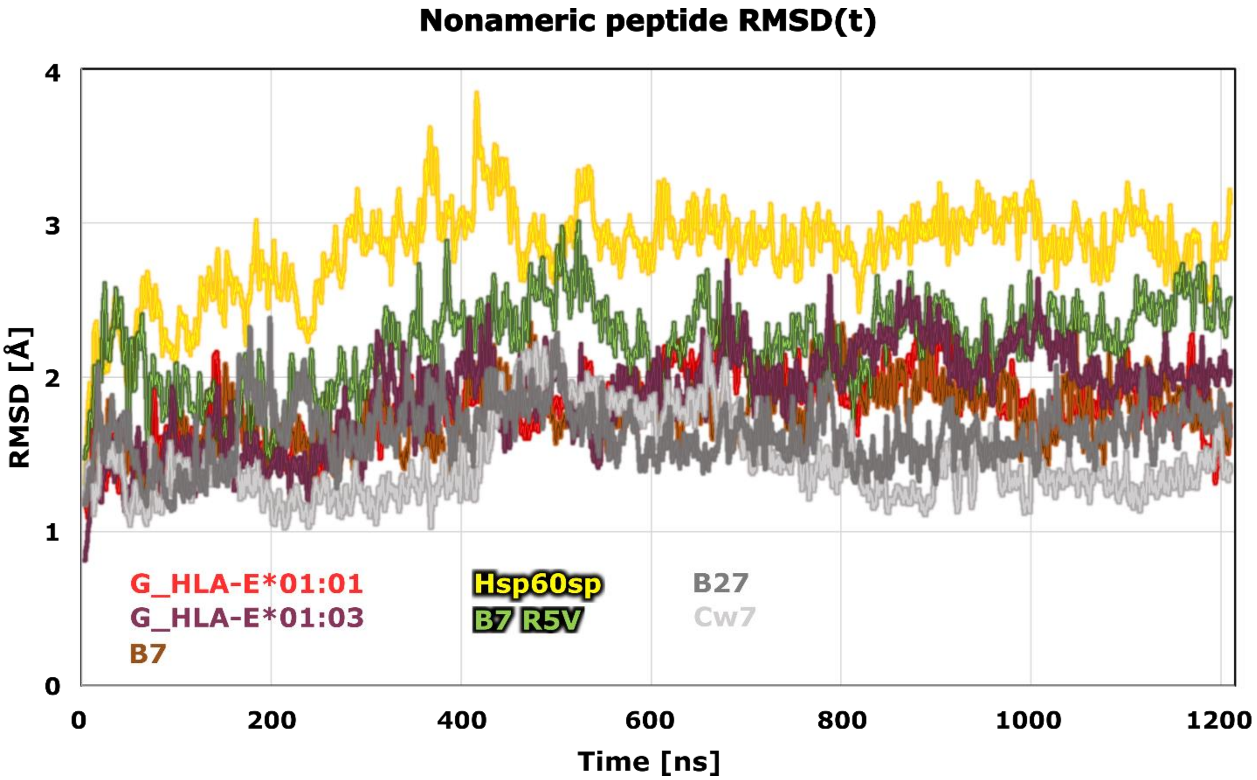

Figure S3. Nonameric peptide's root mean square deviation (RMSD) for models $\mathbf{COM^{+}_{G}}$, $^{allele}\mathbf{COM^{+}_{G}}$, and $\mathbf{COM^{+}_{B7}}$ that provide NK cell protection (red shades), models $\mathbf{COM^{\sim}_{B27}}$ and $\mathbf{COM^{\sim}_{Cw7}}$ with inconclusive (gray shades) and models $\mathbf{COM^{-}_{Hsp60sp}}$ and $\mathbf{COM^{-}_{B7\_R5V}}$ with absent NK cell protection (yellow and green). The moving average with interval 20 was used in data processing.

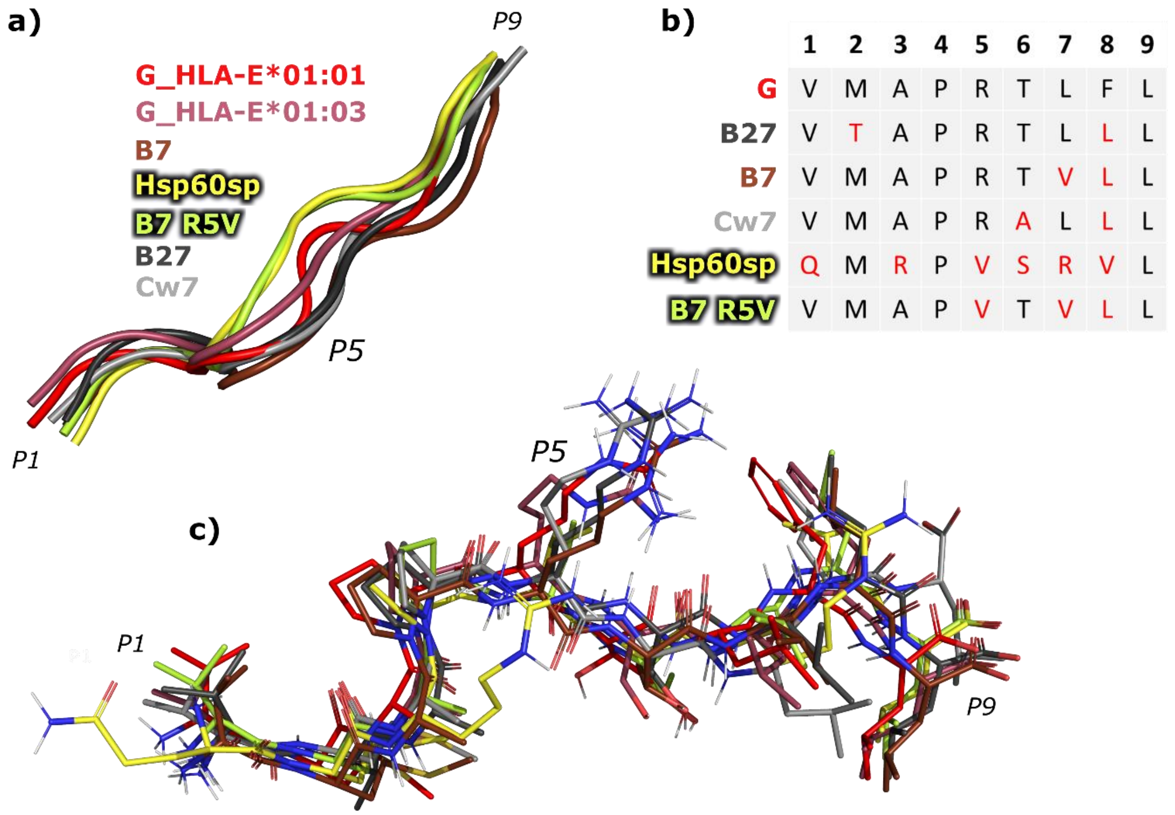

Figure S4. Comparison of nonameric peptide's conformations in the binding pockets of different model complexes. a) Alignment of cartoon representations of nonameric peptides of **COM**$^{+}_{G}$ (red), ^{allele}**COM**$^{+}_{G}$ (pink), **COM**$^{+}_{B7}$ (brown), **COM**$^{-}_{Hsp60sp}$ (yellow), **COM**$^{-}_{B7\_R5V}$ (green), **COM**$^{\sim}_{B27}$ (grey), **COM**$^{\sim}_{Cw7}$ (light grey) shows apparent differences in P5 position. b) Sequence alignment of peptides G, B27, B7, Cw7, Hsp60sp, and B7 R5V, where residues that differ from the peptide G sequence are marked in red. c) Alignment of nonameric peptides G of both HLA-E *01:01 and *01:03 allelic variant models, B7, Cw7, B27, Hsp60sp, and B7 R5V (red, pink, brown, light gray, dark gray, yellow and green respectively) in licorice representation with only polar hydrogens displayed.

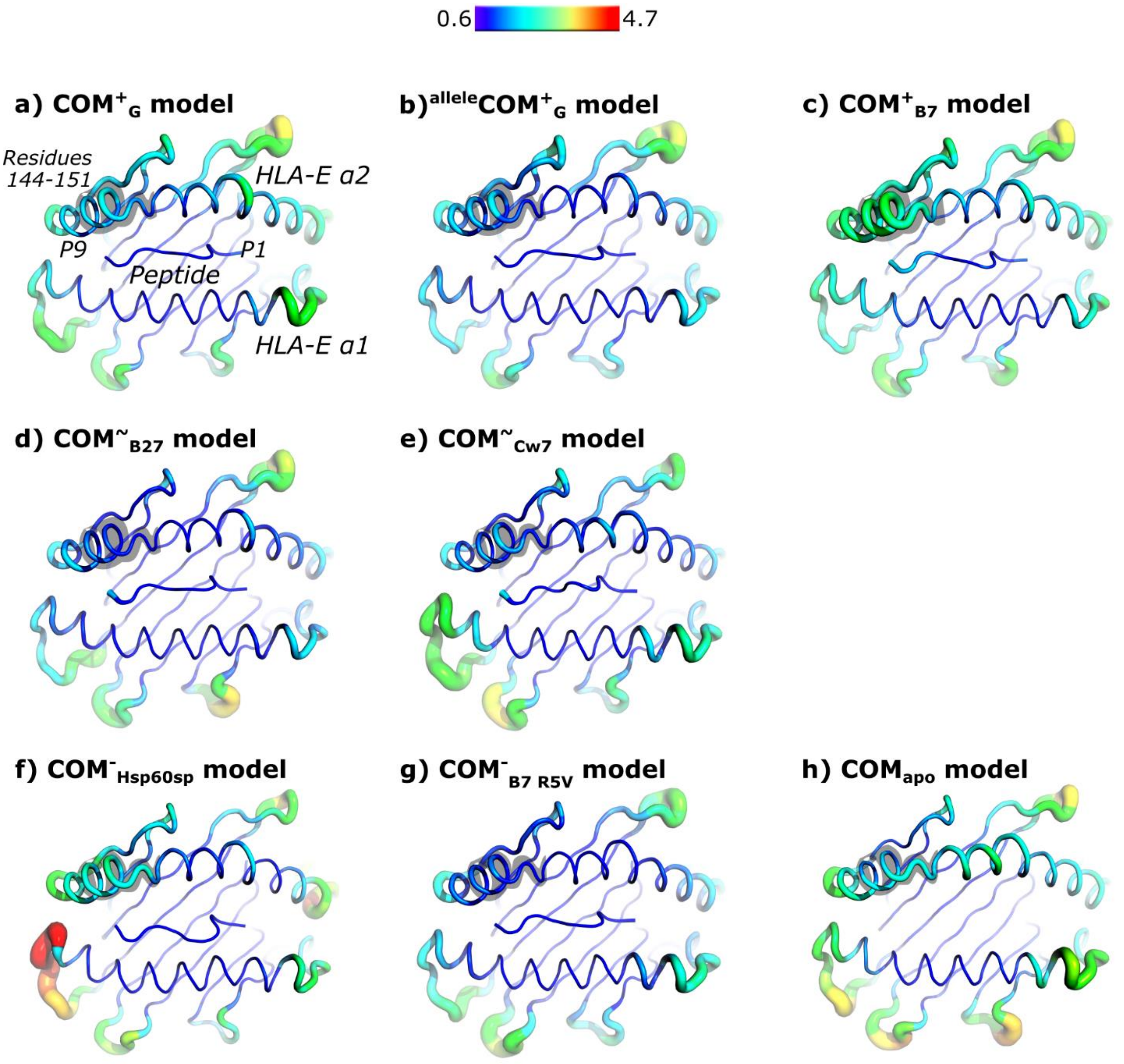

Figure S5. B-factor representation of atomic Root mean square fluctuations (RMSF) of HLA-E α1 and α2 domains and nonameric peptide with shaded area of HLA-E 2α between residues 144 and 151 for a) $\mathbf{COM^{+}_{G}}$, b) $^{allele}\mathbf{COM^{+}_{G}}$, c) $\mathbf{COM^{+}_{B7}}$, d) $\mathbf{COM^{\sim}_{B27}}$**,** e) $\mathbf{COM^{\sim}_{Cw7}}$, f) $\mathbf{COM^{-}_{Hsp60sp}}$**, g)** $\mathbf{COM^{-}_{B7\_R5V}}$ and b) $\mathbf{COM_{apo}}$ models**.**

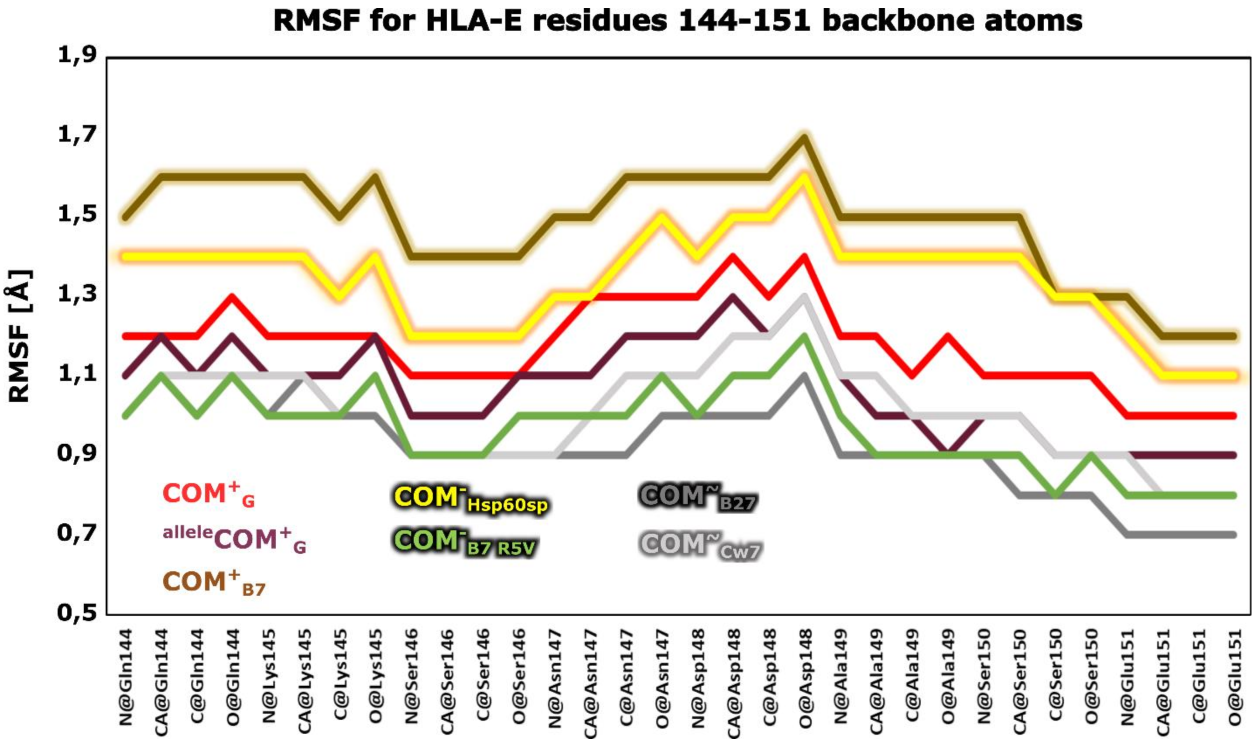

Figure S6. Atomic positional fluctuations (root mean square fluctuations (RMSF)) for backbone HLA-E residues 144-151 for the **COM**$^{+}_{G}$ (red), ^{allele}**COM**$^{+}_{G}$ (violet), **COM**$^{+}_{B7}$ (brown), **COM**$^{\sim}_{B27}$ (dark gray)**,** **COM**$^{\sim}_{Cw7}$ (light gray), **COM**$^{-}_{Hsp60sp}$ (yellow)**,** and **COM**$^{-}_{B7\_R5V}$ (green) models.

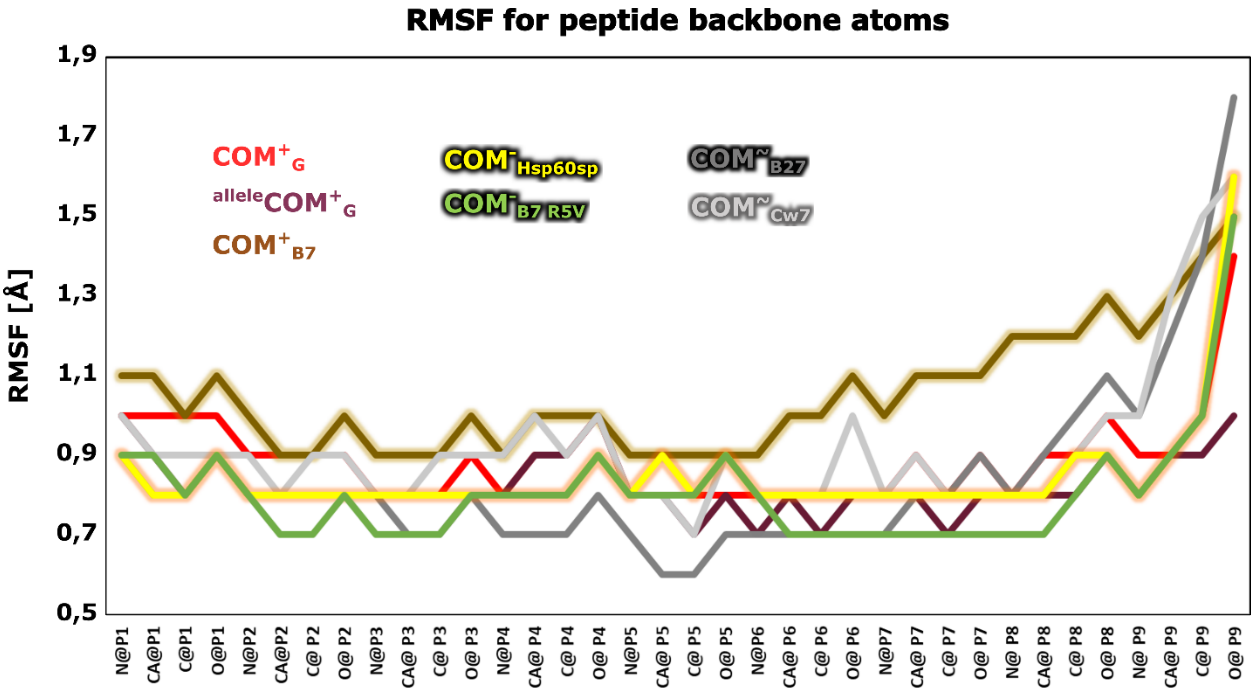

Figure S7. Atomic positional fluctuations (root mean square fluctuations (RMSF)) for peptide backbone atoms for the **COM^{+}$_{G}$** (red), ^{allele}**COM^{+}$_{G}$** (violet), **COM^{+}$_{B7}$** (brown), **COM$^{\sim}$$_{B27}$** (dark gray)**,** **COM$^{\sim}$$_{Cw7}$** (light gray), **COM^{-}$_{Hsp60sp}$** (yellow)**,** and **COM^{-}$_{B7_R5V}$** (green) models.. Peptide is more flexibile at C-terminal side.

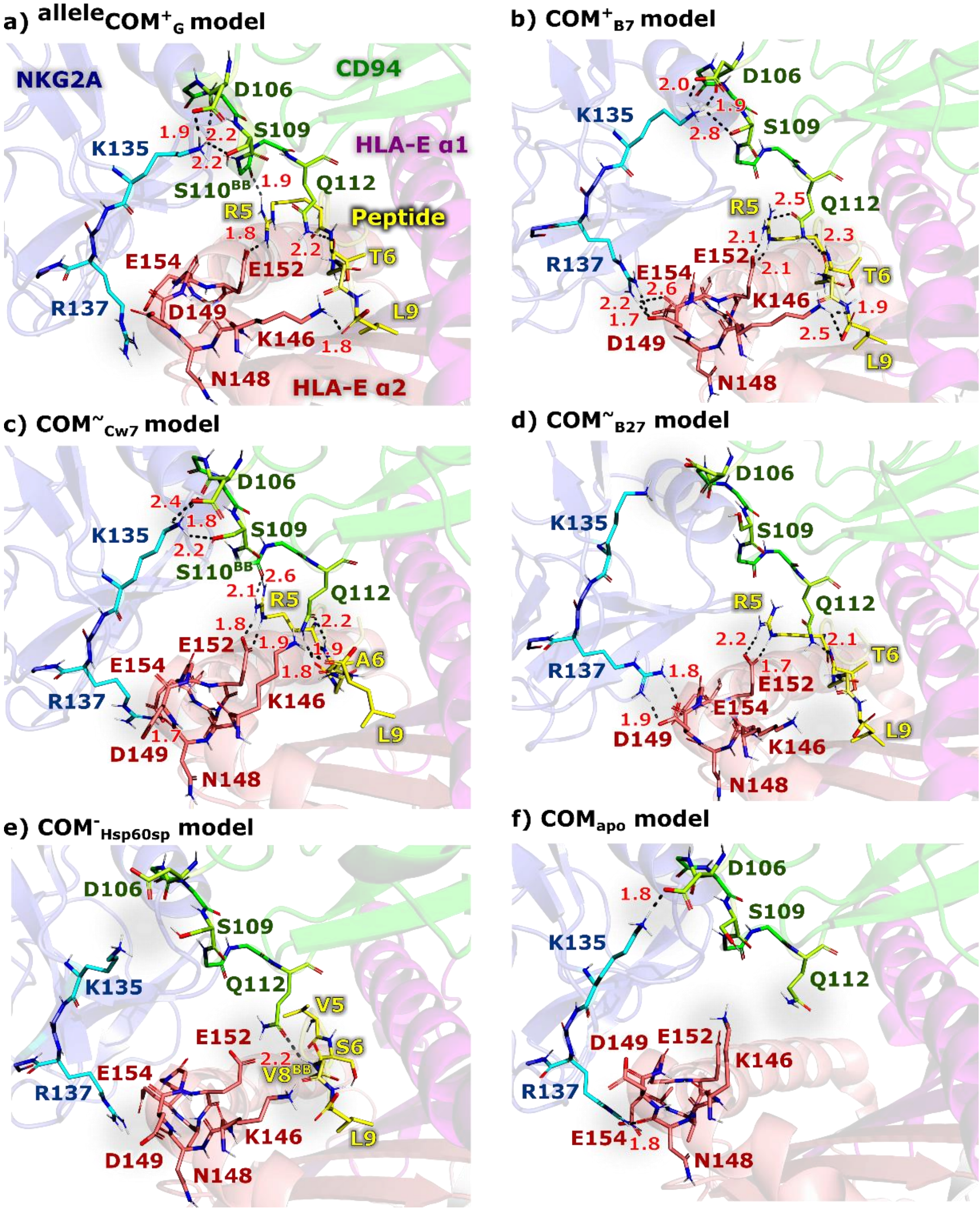

Figure S8. Comparison of predominant hydrogen bonds between NKG2A (cyan; K135), CD94 (green; D106, S109 and Q112), HLA-E (pink; K146, N148, D149, E152 and E154) and peptide (yellow) present in different models according to the most representative clusters of the simulations. Models on the panels a) ^{allele}**COM$^{+}_{G}$** and b) **COM$^{+}_{B7}$** represents ligands which allow NK cell protection, whereas for models c) **COM$^{\sim}_{Cw7}$** and d) **COM$^{\sim}_{B27}$** NK protection is inconclusive, and absent for models e) **COM$^{-}_{Hsp60sp}$** and f) **COM$_{apo}$**. For clarity, only polar hydrogens are shown.

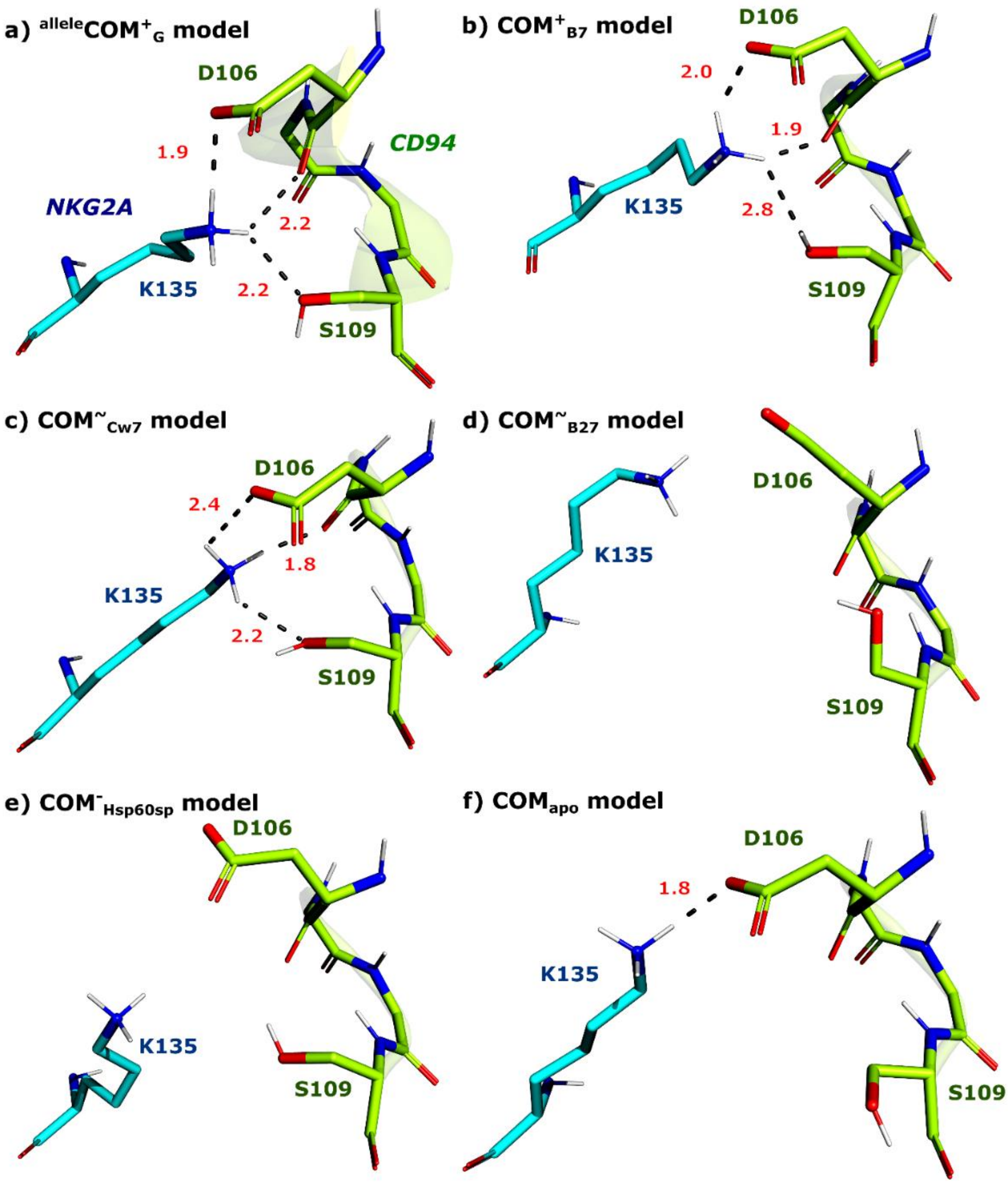

Figure S9. Comparison of predominant hydrogen bonds between NKG2A (cyan; K135) and CD94 (green; D106 and S109) present in different models according to the most representative clusters of the simulations. Models on the panels a) $\mathbf{^{allele}COM^{+}_{G}}$ and b) $\mathbf{COM^{+}_{B7}}$ represents ligands which allow NK cell protection, whereas for models c) $\mathbf{COM^{\sim}_{Cw7}}$ and d) $\mathbf{COM^{\sim}_{B27}}$ NK protection is inconclusive, and absent for models e) $\mathbf{COM^{-}_{Hsp60sp}}$ and f) $\mathbf{COM_{apo}}$. For clarity, unlabeled amino acids are depicted with their backbone atoms only, and only polar hydrogens are shown.

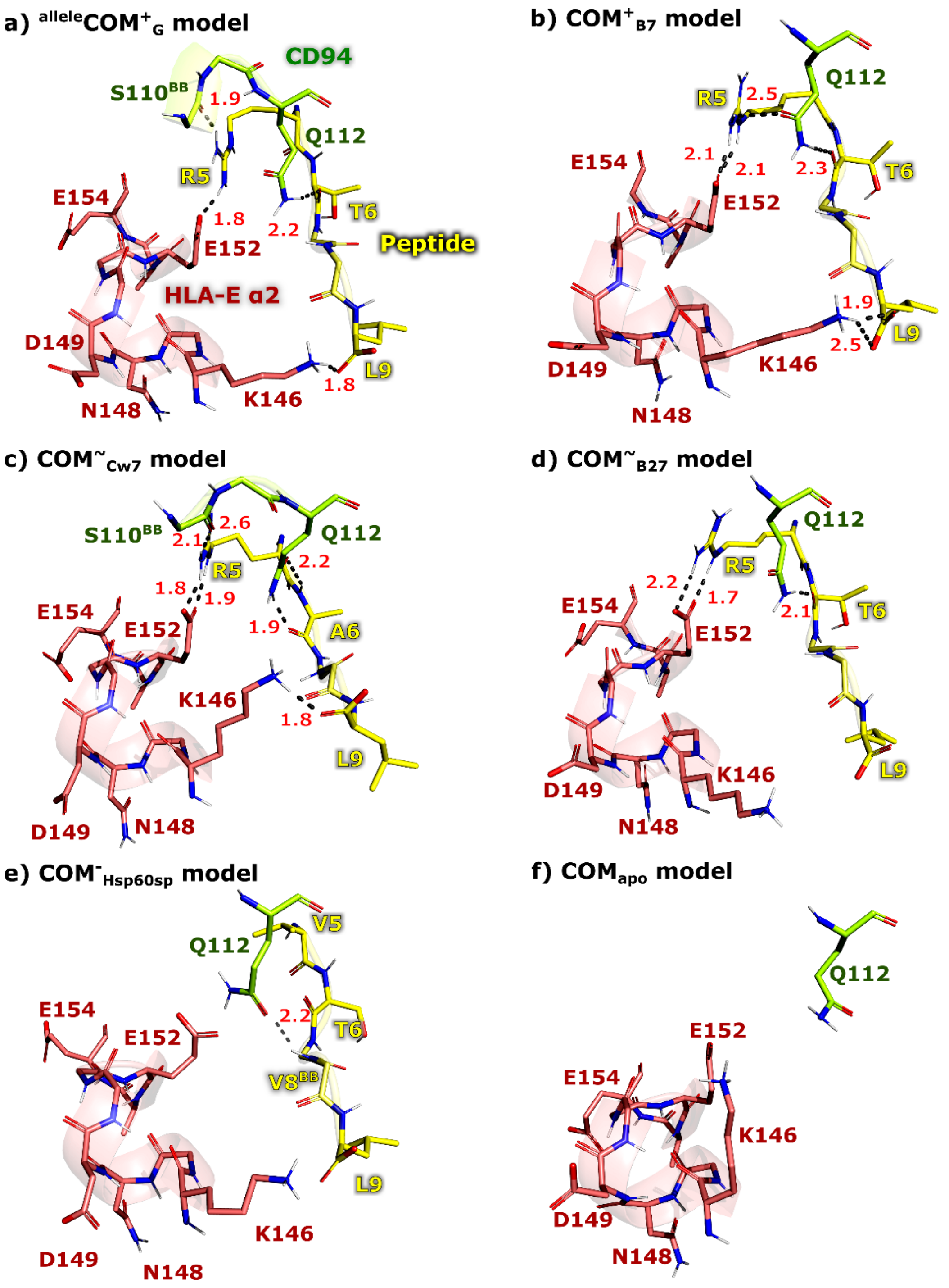

Figure S10. Comparison of predominant hydrogen bonds between nonameric peptide (yellow), CD94 (green: S110 and Q112), and HLA-E (pink; K146, N148, D149, E152 and E154) present in different models according to the most representative clusters of the simulations. Models on the panels a) $^{allele}\mathbf{COM}^{+}_{G}$ and b) $\mathbf{COM}^{+}_{B7}$ represent ligands that provide NK cell protection, whereas for models c) $\mathbf{COM}^{\sim}_{Cw7}$ and d) $\mathbf{COM}^{\sim}_{B27}$ NK protection is inconclusive, and absent for models e) $\mathbf{COM}^{-}_{Hsp60sp}$ and f) $\mathbf{COM}_{apo}$. For clarity, unlabeled amino acids are depicted with their backbone atoms only, and only polar hydrogens are shown.

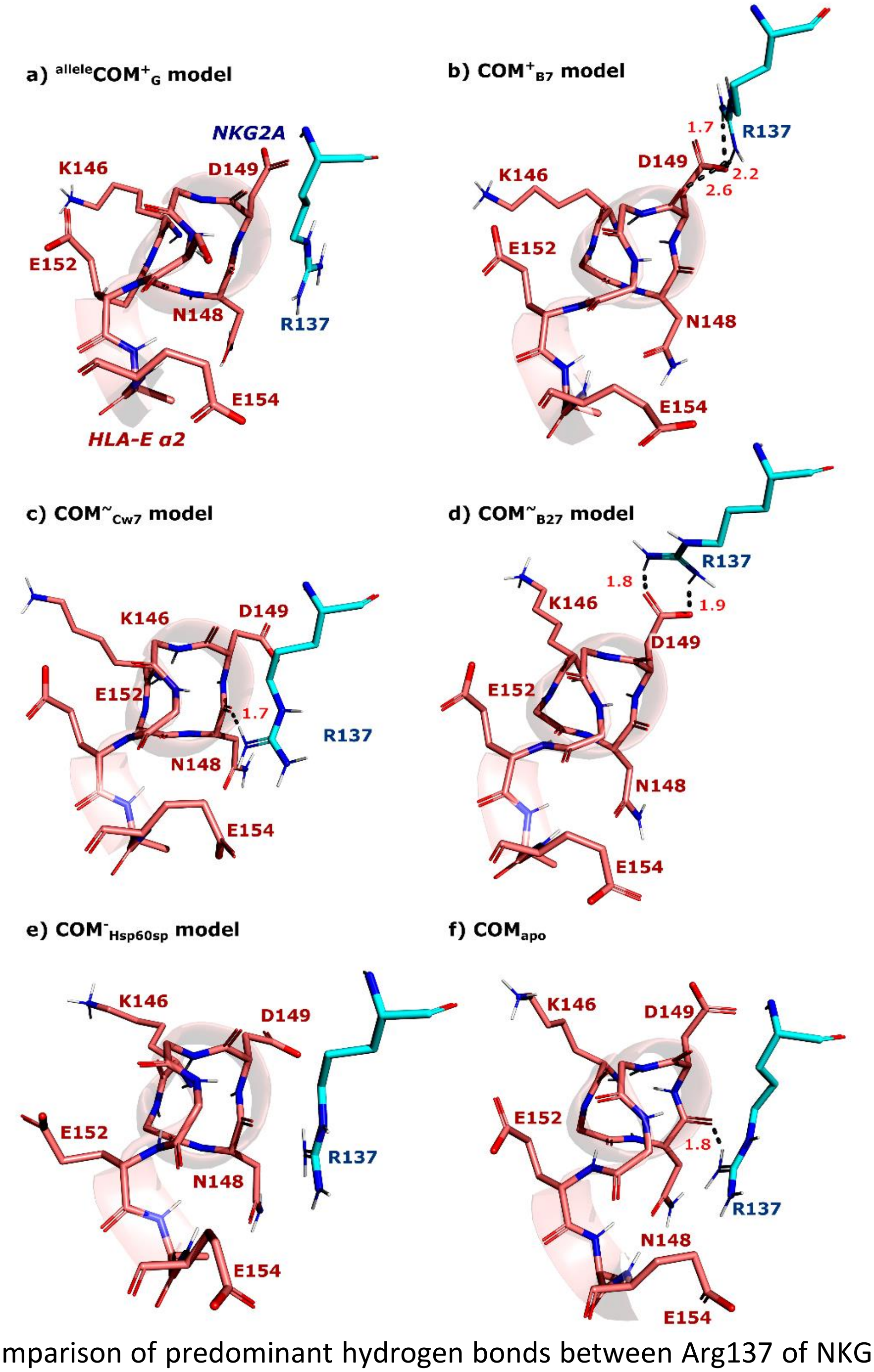

Figure S11. Comparison of predominant hydrogen bonds between Arg137 of NKG2A (cyan) and HLA-E α2 domain (pink; K146, N148, D149, E152 and E154) according to the most representative clusters of the simulations. Models on the panels a) $^{allele}\mathbf{COM}^{+}_{G}$ and b) $\mathbf{COM}^{+}_{B7}$ represent ligands that provide NK cell protection, whereas for models c) $\mathbf{COM}^{\sim}_{Cw7}$ and d) $\mathbf{COM}^{\sim}_{B27}$ NK protection is inconclusive, and absent for models e) $\mathbf{COM}^{-}_{Hsp60sp}$ and f) $\mathbf{COM}_{apo}$. For clarity, unlabeled amino acids are depicted with their backbone atoms only, and only polar hydrogens are shown.

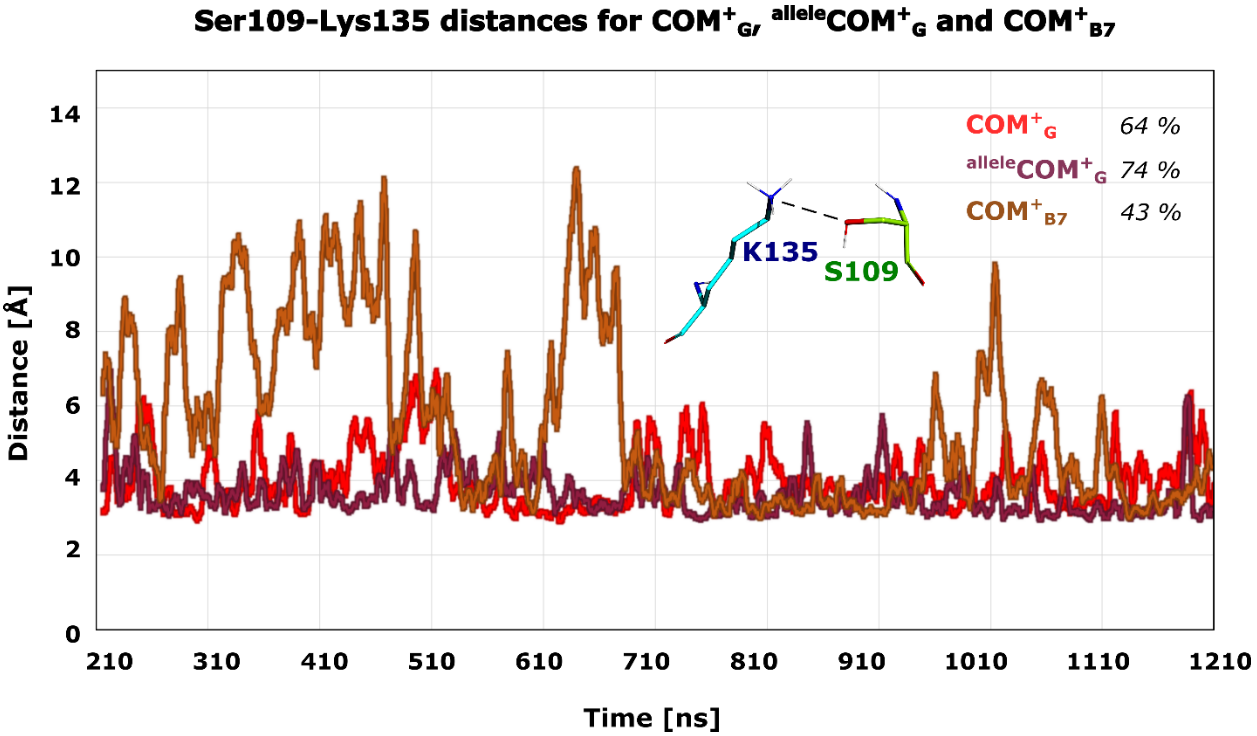

Figure S12. Distances between atoms OG@Ser109 of CD94 (green) and NZ@Lys135 of NKG2A (cyan) for **COM^+_G** (red), **^{allele}COM^+_G** (dark violet) and **COM$^+_{B7}$** (brown) models (providing NK cell protection) vs. simulation time. The percentages next to the model name correspond to the fraction of the equilibrated part of the trajectories where the distance is less than 4 Å. The moving average with interval 20 was used in data processing.

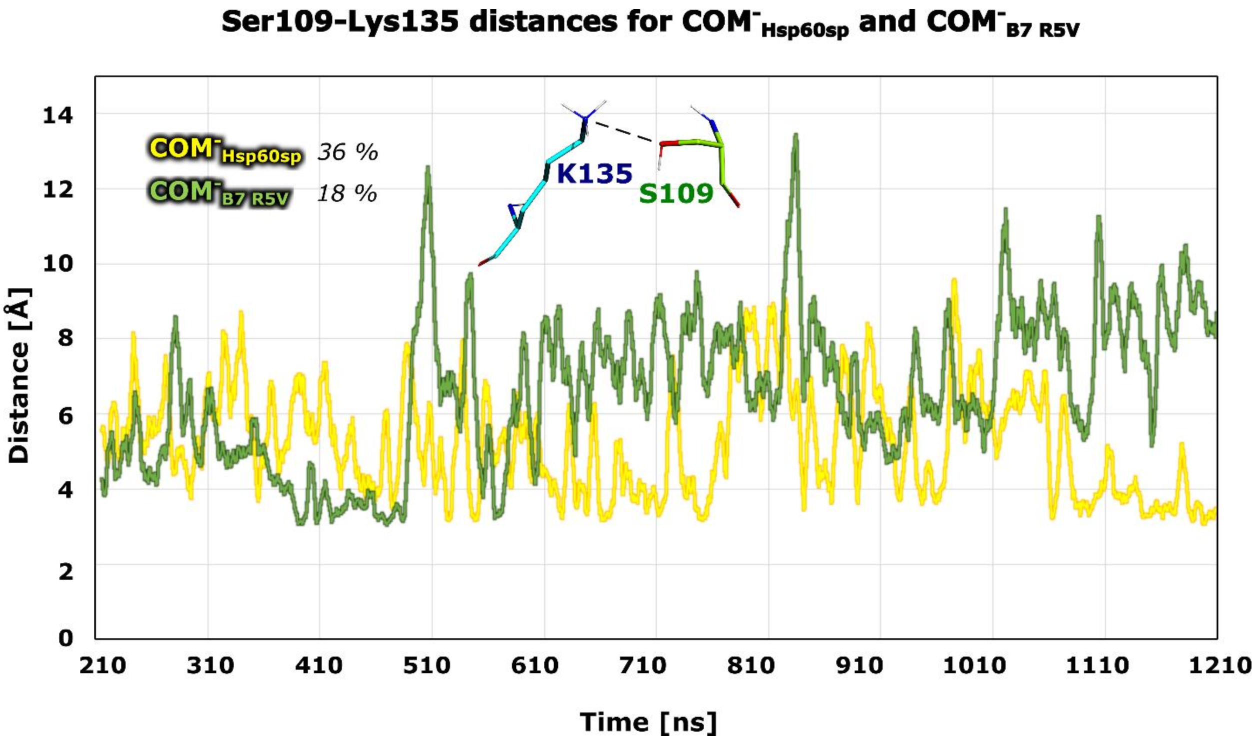

Figure S13. Distances between atoms OG@Ser109 of CD94 (green) and NZ@Lys135 of NKG2A (cyan) for models **COM^{-}$_{Hsp60sp}$** (yellow) and **COM^{-}$_{B7_R5V}$** (green) with absent NK cell protection vs. simulation time. The percentages next to the model name correspond to the fraction of the equilibrated part of the trajectories where the distance is less than 4 Å. The moving average with interval 20 was used in data processing.

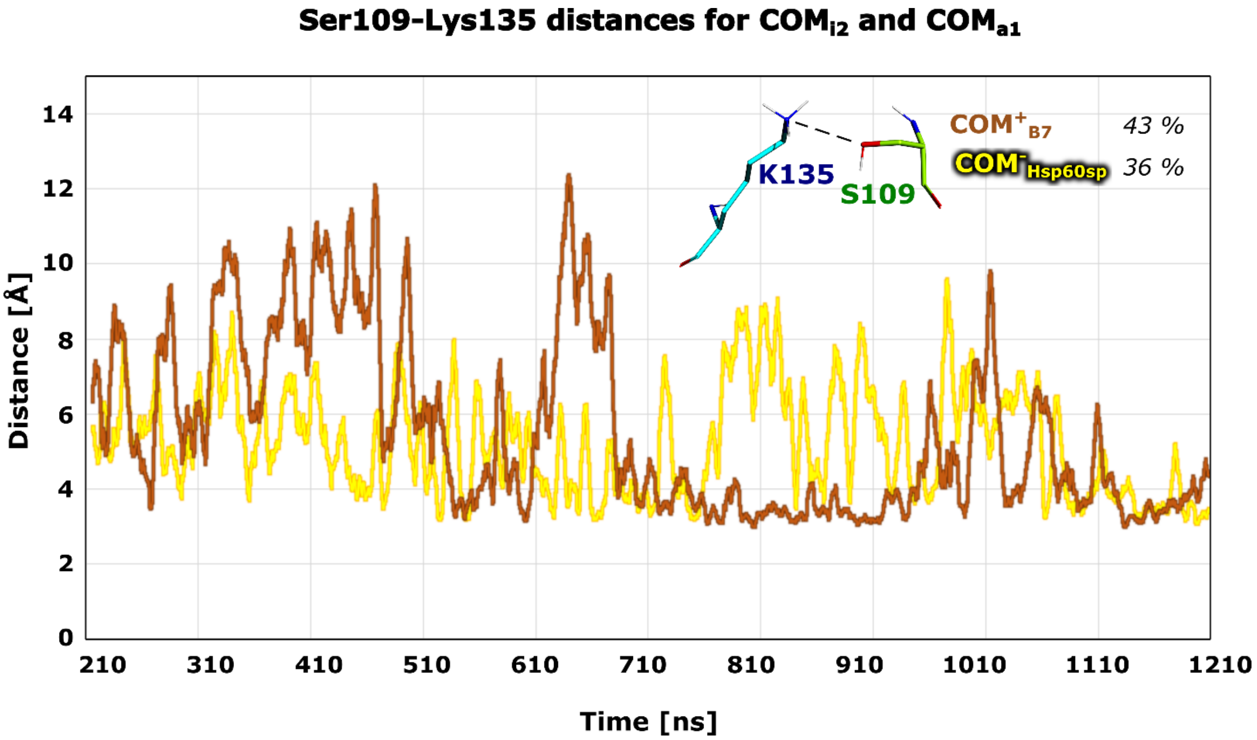

Figure S14. Distances between atoms OG@Ser109 of CD94 (green) and NZ@Lys135 of NKG2A (cyan) for the model **COM^{+}_{B7}** (brown) that provides NK cell protection and model **$COM^{-}_{Hsp60sp}$** (yellow) with absent NK cell protection vs. simulation time. The percentages next to the model name correspond to the fraction of the equilibrated part of the trajectories where the distance is less than 4 Å. The moving average with interval 20 was used in processing the data.

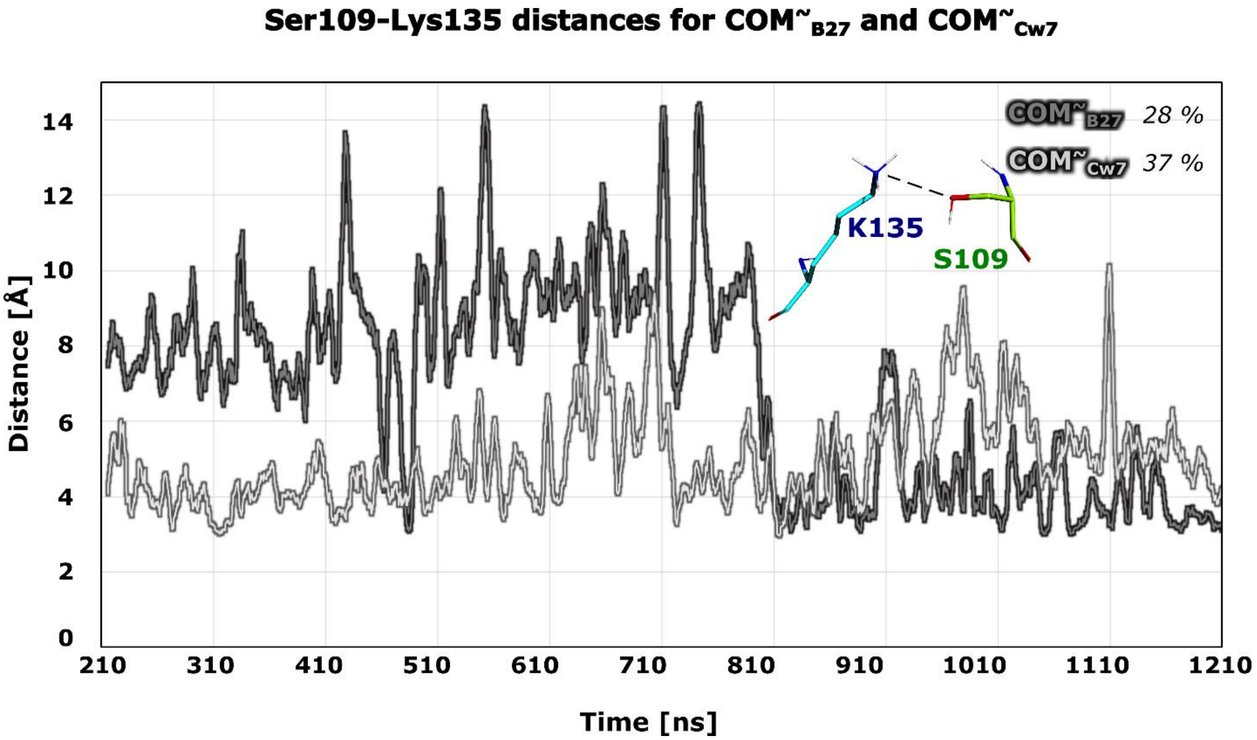

Figure S15. Distances between atoms OG@Ser109 of CD94 (green) and NZ@Lys135 of NKG2A (cyan) for models **COM˜$_{B27}$** (dark gray) and **COM˜$_{Cw7}$** (light gray) with inconclusive NK cell protection vs. simulation time. The percentages next to the model name correspond to the fraction of the equilibrated part of the trajectories where the distance is less than 4 Å. The moving average with interval 20 was used in data processing.

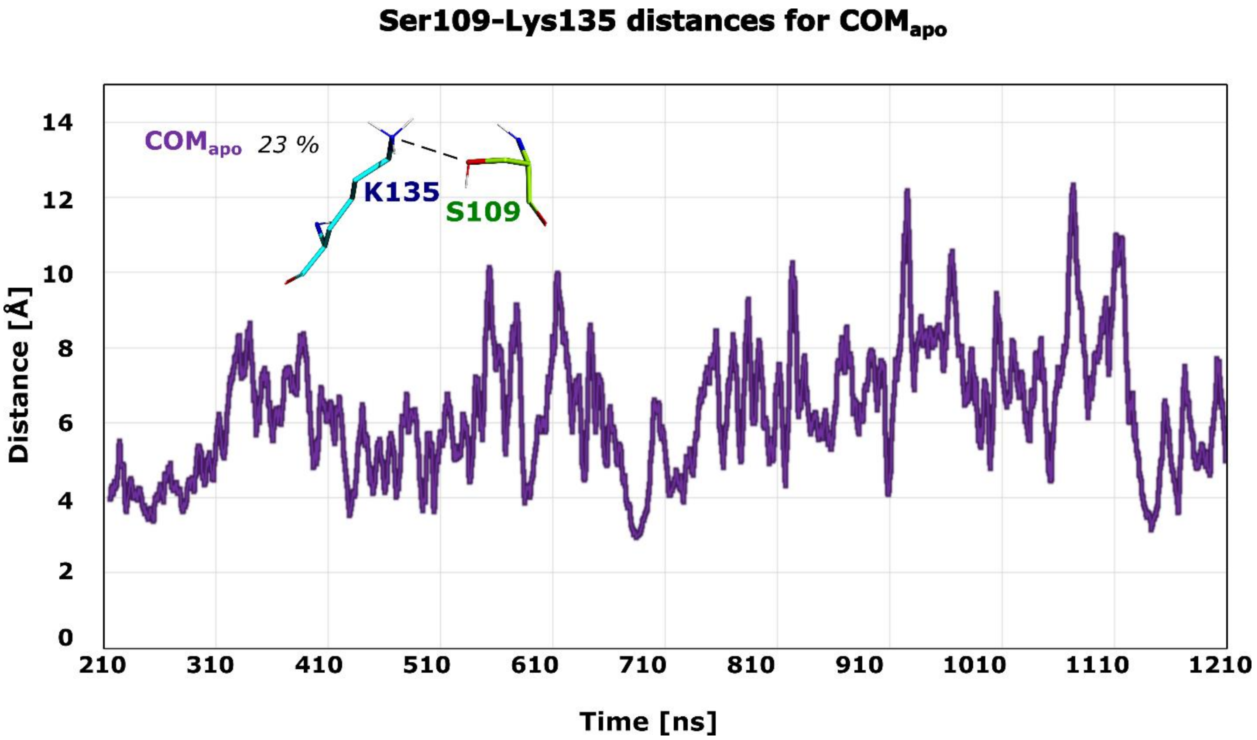

Figure S16. Distances between atoms OG@Ser109 of CD94 (green) and NZ@Lys135 of NKG2A (cyan) for model **COM_{apo}** without nonameric peptide vs. simulation time. The percentage next to the model name corresponds to the fraction of the equilibrated part of the trajectories where the distance is less than 4 Å. The moving average with interval 20 was used in data processing.

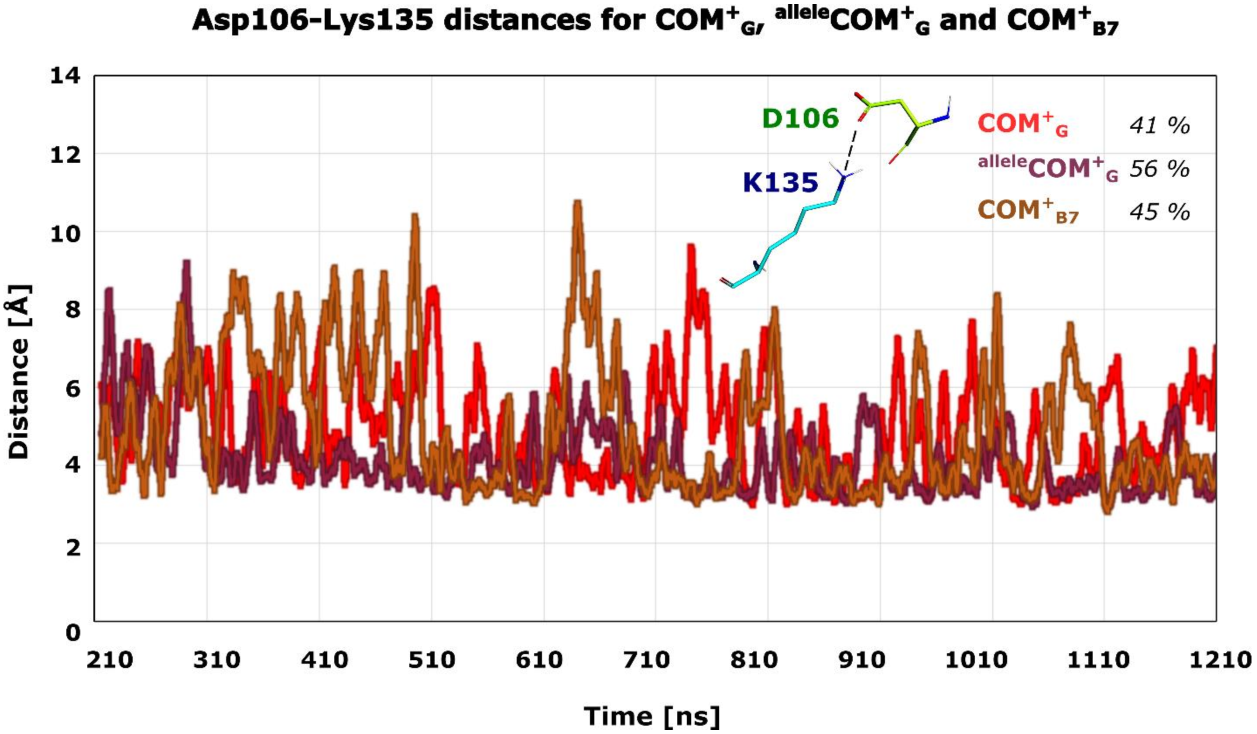

Figure S17. Distances between atom OD1@Asp106 of CD94 (green) and NZ@Lys135 of NKG2A (cyan) for models **COM$^{+}_{G}$** (red), **^{allele}COM$^{+}_{G}$** (dark violet), and **COM$^{+}_{B7}$** (brown) that provide NK cell protection vs. simulation time. The percentages next to the model name correspond to the fraction of the equilibrated part of the trajectories where the distance is less than 4 Å. The moving average with interval 20 was used in data processing.

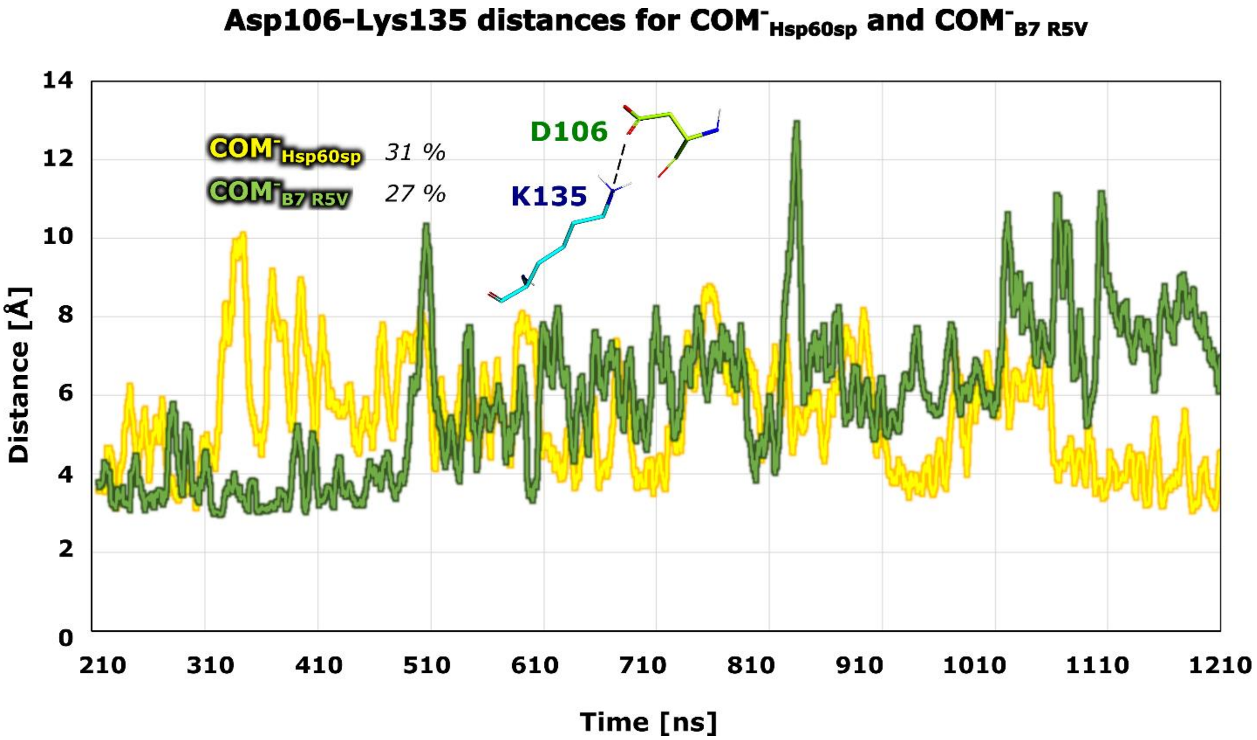

Figure S18. Distances between atoms OD1@Asp106 of CD94 (green) and NZ@Lys135 of NKG2A (cyan) for models **COM**$^{-}$**$_{Hsp60sp}$** (yellow) and **COM**$^{-}$**$_{B7\_R5V}$** (green) with absent NK cell protection vs. simulation time. The percentages next to the model name correspond to the fraction of the equilibrated part of the trajectories where the distance is less than 4 Å. The moving average with interval 20 was used in data processing.

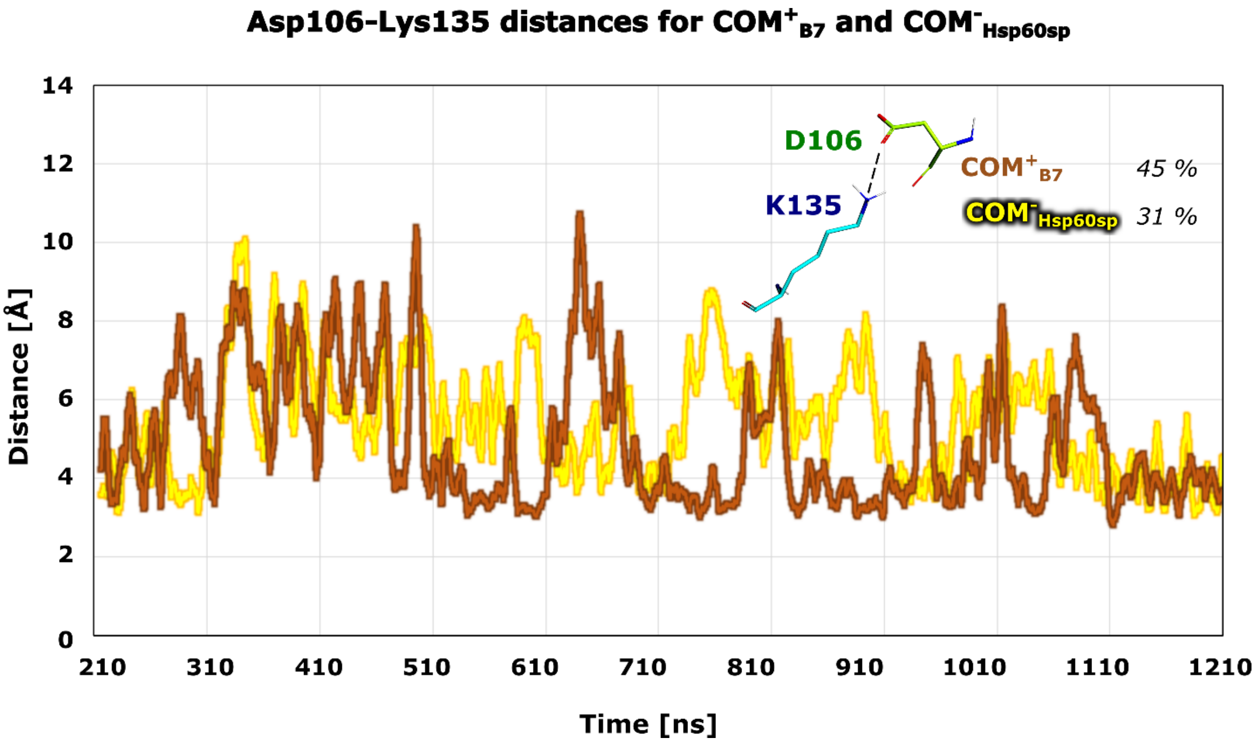

Figure S19. Distances between atoms OD1@Asp106 of CD94 (green) and NZ@Lys135 of NKG2A (cyan) for model **COM^{+}$_{B7}$** (brown) mediating NK cell protection and for model **COM^{-}$_{Hsp60sp}$** (yellow) with absent NK cell protection vs. simulation time. The percentages next to the model name correspond to the fraction of the equilibrated part of the trajectories where the distance is less than 4 Å. The moving average with interval 20 was used in data processing.

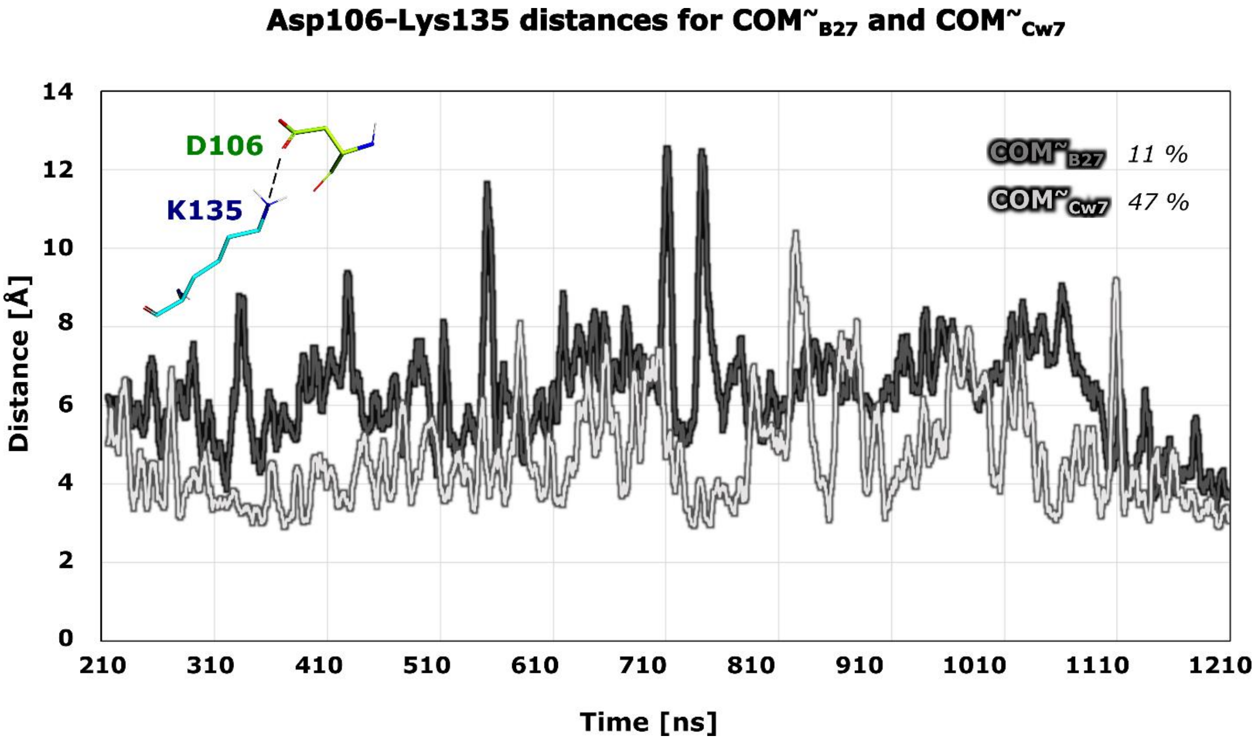

Figure S20. Distances between atoms OD1@Asp106 of CD94 (green) and NZ@Lys135 of NKG2A (cyan) for models **COM˜$_{B27}$** (dark gray) and **COM˜$_{Cw7}$** (light gray) with inconclusive NK cell protection vs. simulation time. The percentages next to the model name correspond to the fraction of the equilibrated part of the trajectories where the distance is less than 4 Å. The moving average with interval 20 was used in data processing.

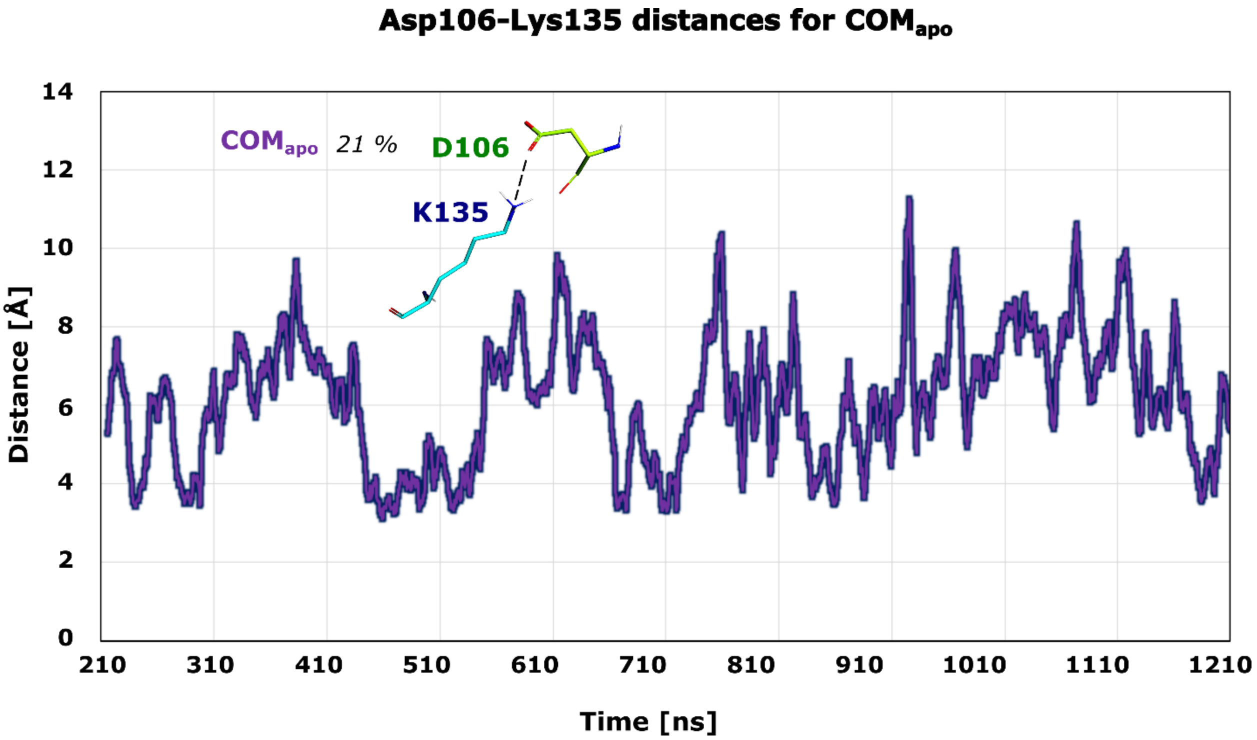

Figure S21. Distances between atoms OD1@Asp106 of CD94 (green) and NZ@Lys135 of NKG2A (cyan) for model **COM$_{apo}$** without nonameric peptide vs. simulation time. The percentage next to the model name corresponds to the fraction of the equilibrated part of the trajectories where the distance is less than 4 Å. The moving average with interval 20 was used in data processing.

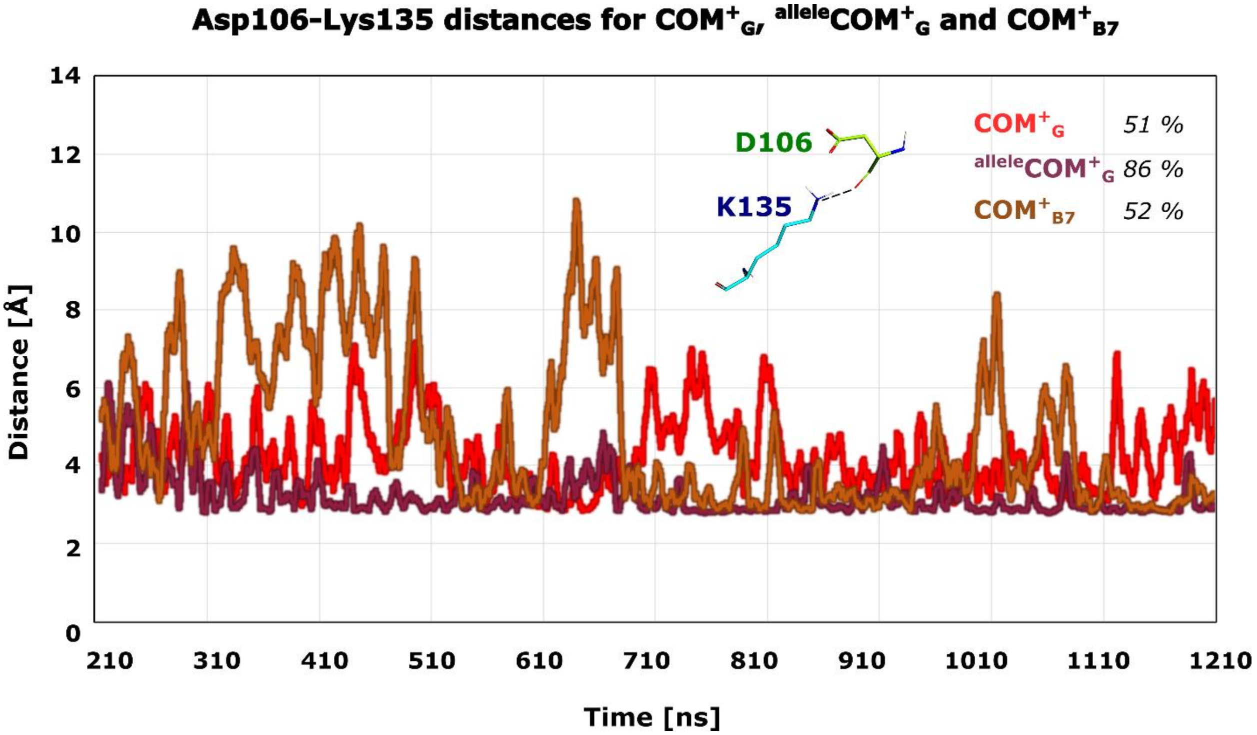

Figure S22. Distances between atoms O@Asp106 of CD94 (green) and NZ@Lys135 of NKG2A (cyan) for models **COM^{+}_{G}** (red), **$^{allele}COM^{+}_{G}$** (dark violet), and **COM^{+}_{B7}** (brown) exhibiting NK cell protection vs. simulation time. The percentages next to the model name correspond to the fraction of the equilibrated part of the trajectories where the distance is less than 4 Å. The moving average with interval 20 was used in data processing.

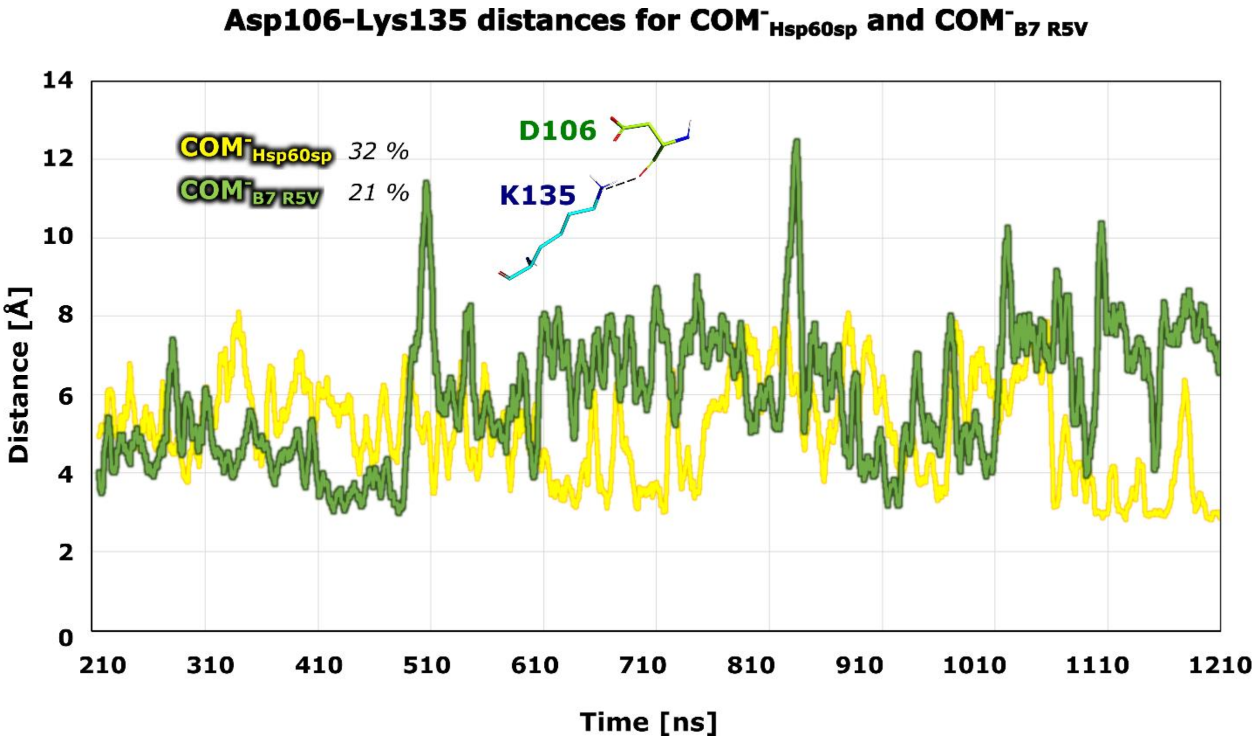

Figure S23. Distances between atoms O@Asp106 of CD94 (green) and NZ@Lys135 of NKG2A (cyan) for models **COM$^-$$_{Hsp60sp}$** (yellow) and **COM$^-$$_{B7_R5V}$** (green) with absent NK cell protection vs. simulation time. The percentages next to the model name correspond to the fraction of the equilibrated part of the trajectories where the distance is less than 4 Å. The moving average with interval 20 was used in data processing.

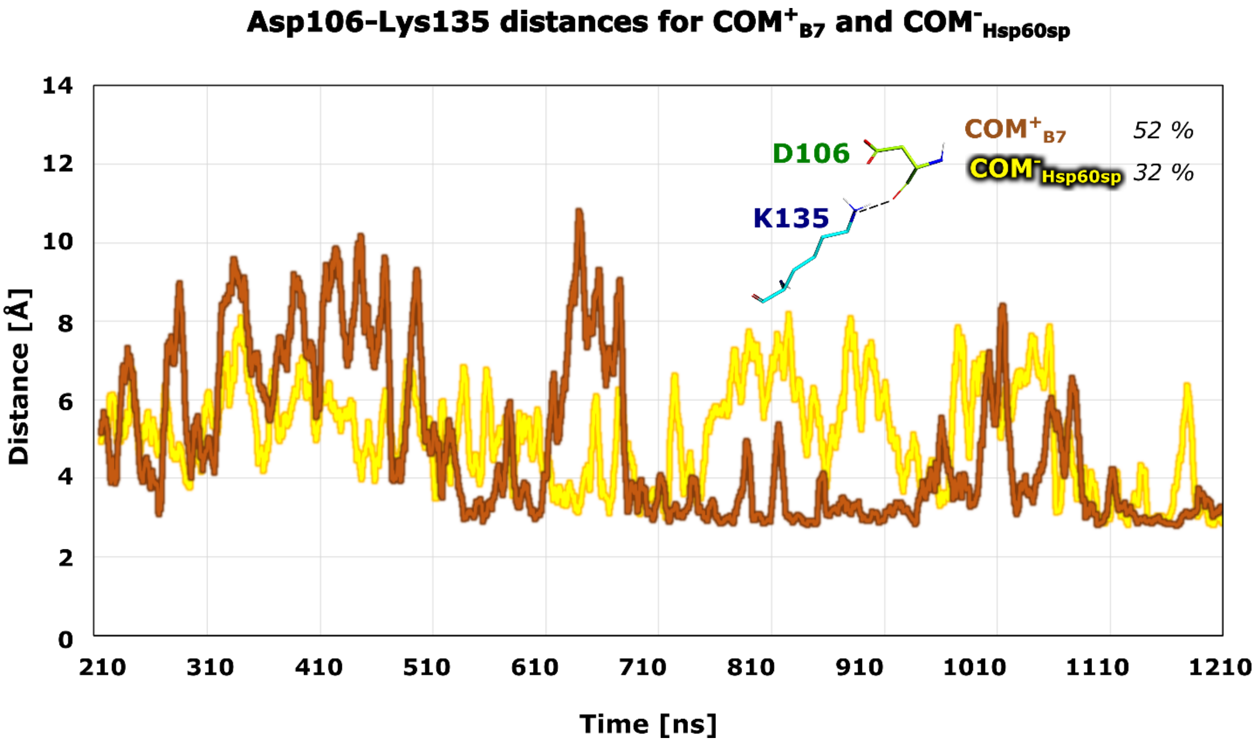

Figure S24. Distances between atoms O@Asp106 of CD94 (green) and NZ@Lys135 of NKG2A (cyan) for model **COM**$^{+}$$_{\mathbf{B7}}$ (brown) providing NK cell protection and model **COM**$^{-}$$_{\mathbf{Hsp60sp}}$ (yellow) with absent NK cell protection vs. simulation time. The percentages next to the model name correspond to the fraction of the equilibrated part of the trajectories where the distance is less than 4 Å. The moving average with interval 20 was used in data processing.

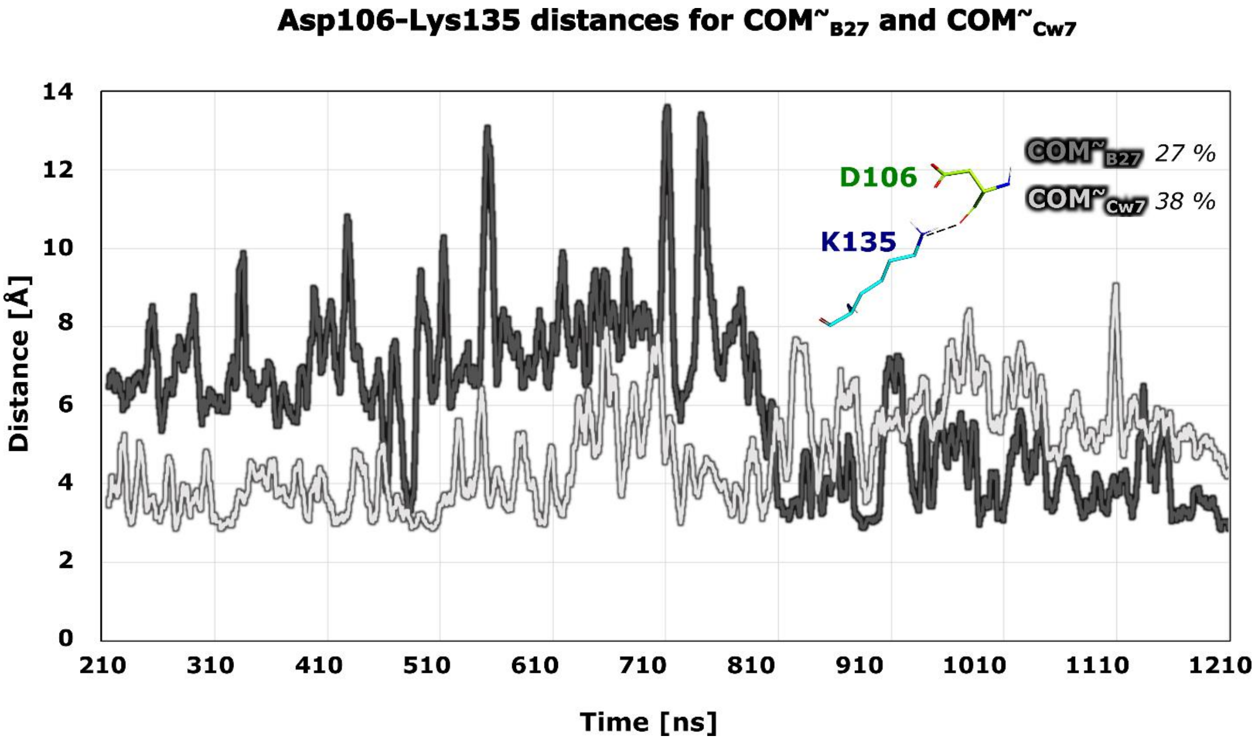

Figure S25. Distances between atoms O@Asp106 of CD94 (green) and NZ@Lys135 of NKG2A (cyan) for models **COM˜$_{B27}$** (dark gray) and **COM˜$_{Cw7}$** (light gray) with inconclusive NK cell protection vs. simulation time. The percentages next to the model name correspond to the fraction of the equilibrated part of the trajectories where the distance is less than 4 Å. The moving average with interval 20 was used in data processing.

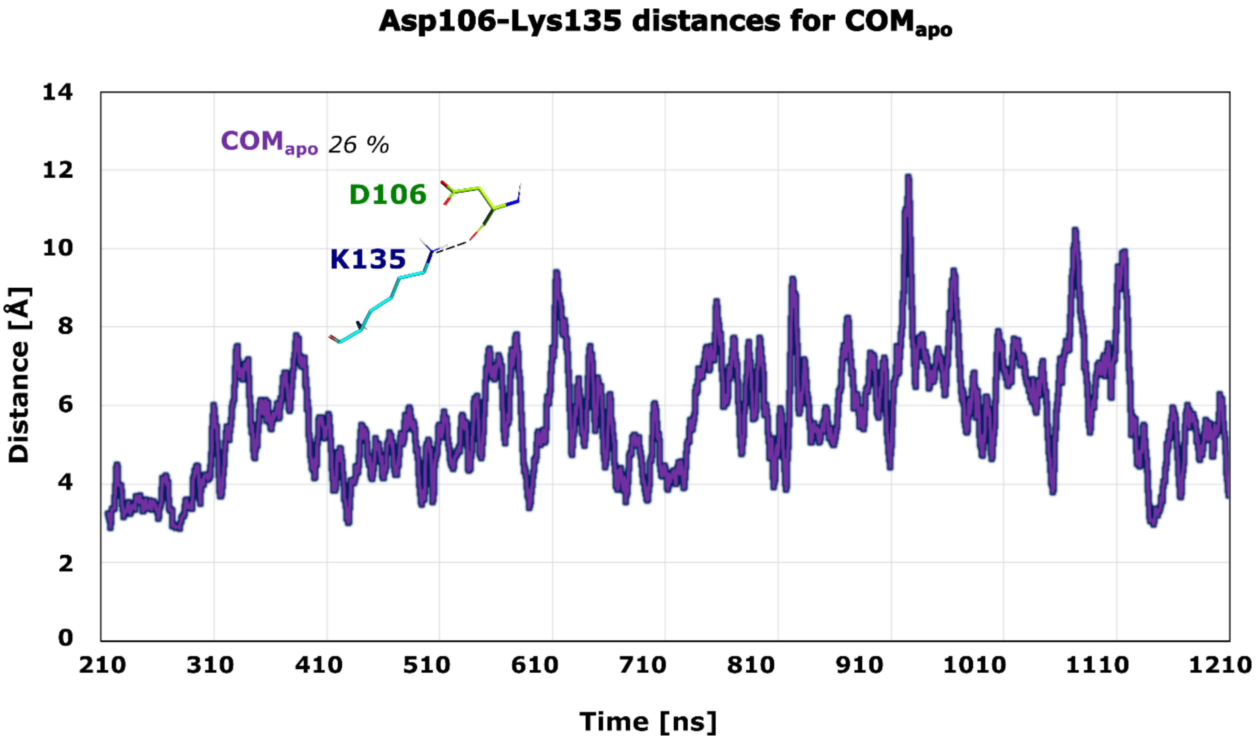

Figure S26. Distances between atoms O@Asp106 of CD94 (green) and NZ@Lys135 of NKG2A (cyan) for model **COM_{apo}** without nonameric peptide vs. simulation time. The percentage next to the model name corresponds to the fraction of the equilibrated part of the trajectories where the distance is less than 4 Å. The moving average with interval 20 was used in data processing.

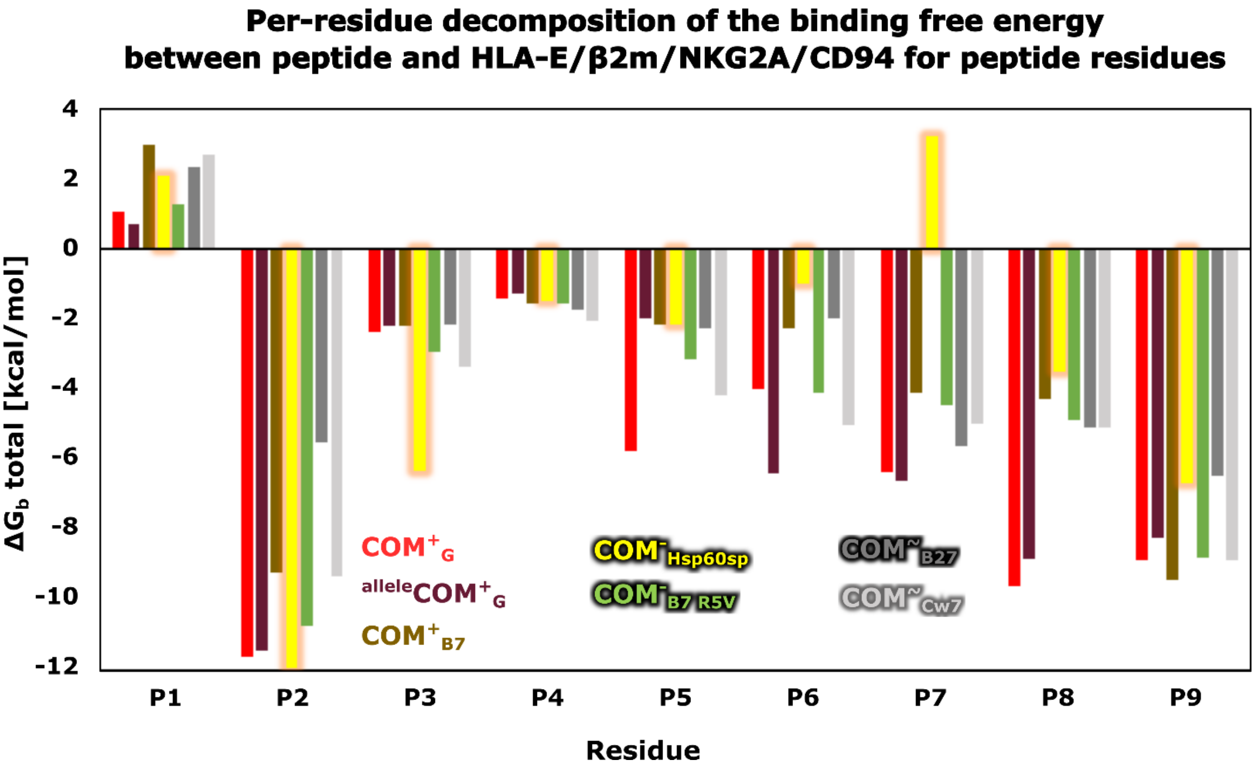

Figure S27. Peptide per-residue decomposition of the Molecular Mechanics-Generalized Born Surface area (MM-GBSA) binding free energies (ΔG_b) between the peptide and the HLA-E/β2m/NKG2A/CD94 calculated over a time interval between 600 and 900 ns of the MD trajectory in the production run for the simulated models ^{allele}**COM**$^{+}_{G}$, **COM**$^{+}_{B7}$, **COM**$^{\sim}_{B27}$, **COM**$^{\sim}_{Cw7}$, **COM**$^{-}_{Hsp60sp}$, and **COM**$^{-}_{B7\_R5V}$.

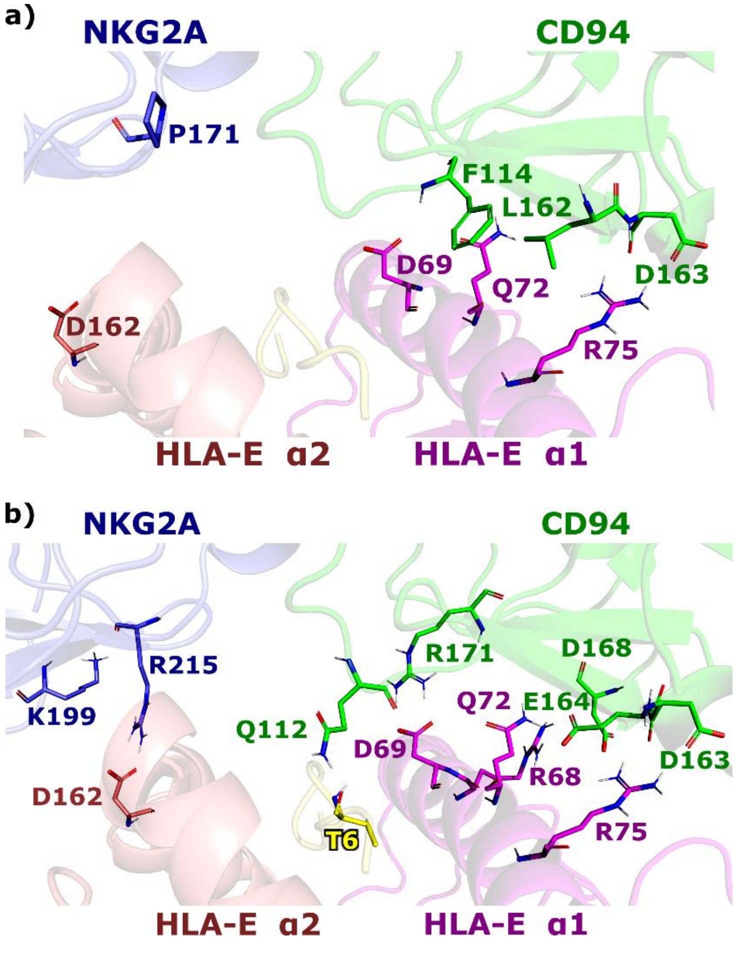

Figure S28. Interacting amino-acids with greatest contribution to the binding free energy between ligand (HLA-E/β2m/peptide) and receptor (NKG2A/CD94), determined with MM-GBSA method with a) per-residue decomposition and b) pairwise decomposition. Depicted with licorice and new cartoon representation. For clarity, only polar hydrogens are presented.

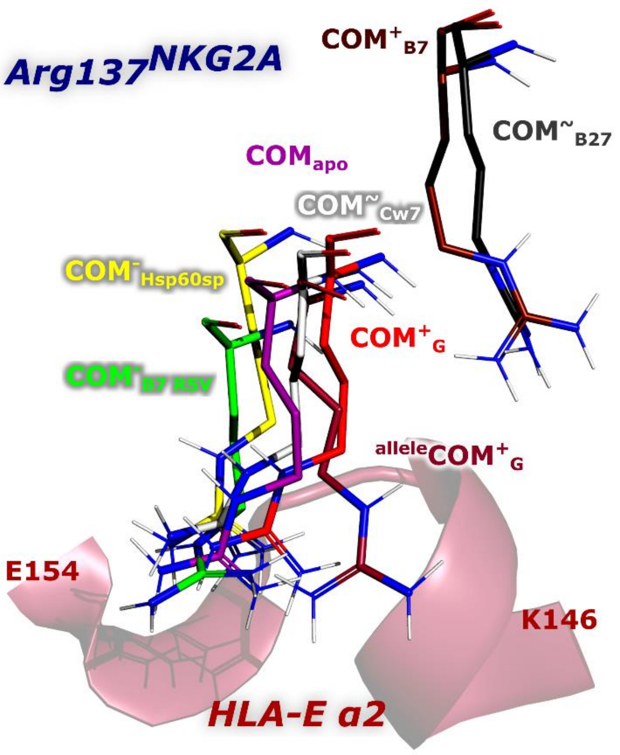

Figure S29. Alignment of Arg137^{NKG2A} and its position according to HLA-E α2 domain. For better visibility, only polar hydrogens are shown, meanwhile HLA-E α2 domain is presented as a single cartoon representation.

Supporting Tables

Table S1. Occurrence of hydrogen (H)-bonds between the selected residues of immune complex reported for the models $\mathbf{COM^{+}_{G}}$, $\mathbf{^{allele}COM^{+}_{G}}$, $\mathbf{COM^{+}_{B7}}$, $\mathbf{COM^{-}_{Hsp60sp}}$, $\mathbf{COM^{-}_{B7\_R5V}}$, $\mathbf{COM^{\sim}_{B27}}$, and $\mathbf{COM^{\sim}_{Cw7}}$.

**Interacting amino acids**		**Models**							
**Acceptor**	**Donor**	COM^{+}_{G}	$^{allele}COM^{+}_{G}$	COM^{+}_{B7}	$COM^{-}_{Hsp60sp}$	$COM^{-}_{B7\_R5V}$	COM_{apo}	$COM^{\sim}_{B27}$	$COM^{\sim}_{Cw7}$
$Asp106^{CD94}$	$Lys135^{NKG2A}$	•	•	•			•		•
$Ser109^{CD94}$	$Lys135^{NKG2A}$	•	•	•					•
$Glu152^{HLA\text{-}E}$	P5	•	•	•				•	•
P6	$Gln112^{CD94}$	•	•	•		•		•	•
$Gln112^{CD94}$	P8				•			•	
P9	$Lys146^{HLA\text{-}E}$	•	•	•		•			•

Table S2. Molecular mechanics-generalized born surface area (MM-GBSA) binding free energies (ΔG_b) between peptide and HLA-E/β2m/NKG2A/CD94 and its pair-wise decomposition (kcal/mol) calculated over time interval between 600 and 900 ns of the production MD trajectory for **COM$^{+}_{B7}$, COM$^{\sim}_{B27}$, COM$^{\sim}_{Cw7}$, COM$^{-}_{Hsp60sp}$,** and **COM$^{-}_{B7_R5V}$** models. The table lists five major interactions between peptide and receptor (NKG2A/CD94) and HLA-E residues.

**^{allele}COM$^{+}_{G}$ ΔG_b -120 ± 9.0 [kcal/mol]**		
**Res 1**	**Res 2**	**ΔG_b total [kcal/mol]**
CD94		
Gln112	Thr^{P6}	-4.0 ± 0.4
Gln112	Phe^{P8}	-3.1 ± 0.5
Ser110	Arg^{P5}	-3.1 ± 1.4
Gln112	Arg^{P5}	-1.1 ± 0.8
Asn158	Phe^{P8}	-1.6 ± 0.4
HLA-E		
Glu63	Val^{P1}	-25.1 ± 2.9
Glu152	Arg^{P5}	-16.1 ± 4.2
Gln156	Arg^{P5}	-11.3 ± 1.1
Lys146	Leu^{P9}	-11.0 ± 6.6
Ser143	Leu^{P9}	-6.8 ± 1.3

**COM$^{+}_{B7}$ ΔG_b -89.3 ± 8.4 [kcal/mol]**		
**Res 1**	**Res 2**	**ΔG_b total [kcal/mol]**
CD94		
Gln112	Thr^{P6}	-2.9 ± 1.0
Gln112	Leu^{P8}	-1.9 ± 0.5
Gln112	Arg^{P5}	-2.0 ± 1.1
Ser110	Arg^{P5}	-2.5 ± 2.6
NKG2A		
Pro171	Arg^{P5}	-1.3 ± 0.3
HLA-E		
Glu63	Val^{P1}	-21.4 ± 3.7
Glu152	Arg^{P5}	-16.6 ± 3.8
Lys146	Leu^{P9}	-15.0 ± 4.3
Ser143	Leu^{P9}	-7.0 ± 1.1
Tyr84	Leu^{P9}	-6.2 ± 1.2

**COM$^{\sim}_{B27}$ ΔG_b -77.8 ± 9.4 [kcal/mol]**		
**Res 1**	**Res 2**	**ΔG_b total [kcal/mol]**
CD94		
Gln112	Thr^{P6}	-2.6 ± 1.4
Gln112	Leu^{P8}	-1.8 ± 0.7
Gln112	Arg^{P5}	-1.8 ± 0.7
NKG2A		
Pro171	Arg^{P5}	-0.9 ± 0.3
Val213	Arg^{P5}	-1.1 ± 0.3
HLA-E		
Glu63	Val^{P1}	-20.3 ± 5.0
Glu152	Arg^{P5}	-16.9 ± 1.8
Lys146	Leu^{P9}	-9.7 ± 6.4
Tyr7	Val^{P1}	-6.0 ± 1.4
Tyr84	Leu^{P9}	-5.2 ± 1.7

**COM$^{\sim}_{Cw7}$ ΔG_b -94.9 ± 8.5 [kcal/mol]**		
**Res 1**	**Res 2**	**ΔG_b total [kcal/mol]**
CD94		
Gln112	Ala^{P6}	-4.4 ± 0.3
Gln112	Leu^{P8}	-2.6 ± 0.4
Gln112	Arg^{P5}	-2.6 ± 1.0
Ser110	Arg^{P5}	-5.2 ± 1.4
NKG2A		
Pro171	Arg^{P5}	-1.6 ± 0.4
HLA-E		
Glu63	Val^{P1}	-21.0 ± 4.3
Glu152	Arg^{P5}	-16.3 ± 1.9
Lys146	Leu^{P9}	-14.1 ± 5.1
Ser143	Leu^{P9}	-6.8 ± 1.1
Trp167	Val^{P1}	-6.7 ± 1.7

**COM$^{-}_{Hsp60sp}$ ΔG_b -82.1 ± 8.3 [kcal/mol]**		
**Res 1**	**Res 2**	**ΔG_b total [kcal/mol]**
CD94		
Gln112	Ser^{P6}	-0.5 ± 0.7
Gln112	Val^{P8}	-1.2 ± 1.1
Gln112	Val^{P5}	-1.4 ± 0.0
Gln112	Arg^{P7}	-1.3 ± 2.8
Gln113	Val^{P5}	-1.0 ± 0.3
HLA-E		
Glu63	Gln^{P1}	-23.1 ± 2.3
Glu152	Arg^{P3}	-15.7 ± 2.3
Lys146	Leu^{P9}	-10.1 ± 4.9
Gln156	Arg^{P3}	-6.9 ± 0.9
Ser143	Leu^{P9}	-6.3 ± 1.4

Table S3. Molecular mechanics-generalized born surface area (MM-GBSA) binding free energies (ΔG_b) between peptide and HLA-E/β2m/NKG2A/CD94 and its per-residue decomposition (kcal/mol) calculated over time interval between 600 and 900 ns of the MD trajectory in the production run for **$^{allele}COM^{+}_{G}$, COM^{+}_{B7}, $COM^{\sim}_{B27}$, $COM^{\sim}_{Cw7}$, $COM^{-}_{Hsp60sp}$,** and **$COM^{-}_{B7\_R5V}$** models. In the table are listed energies for peptide residues.

**$^{allele}COM^{+}_{G}$ ΔG_b -120 ± 9.0 [kcal/mol]**	
**Residue**	**ΔG_b total [kcal/mol]**
Met^{P2}	-11.5 ± 0.9
Phe^{P8}	-8.8 ± 1.3
Leu^{P9}	-8.2 ± 3.5
Leu^{P7}	-6.6 ± 0.9
Thr^{P6}	-6.4 ± 1.0
Ala^{P3}	-2.2 ± 0.5
Arg^{P5}	-2.0 ± 2.2
Pro^{P4}	-1.3 ± 0.5
Val^{P1}	0.7 ± 1.7

**COM^{+}_{B7} ΔG_b -89.3 ± 8.4 [kcal/mol]**	
**Residue**	**ΔG_b total [kcal/mol]**
Leu^{P9}	-9.4 ± 2.5
Met^{P2}	-9.2 ± 1.0
Leu^{P8}	-4.3 ± 0.9
Val^{P7}	-4.1 ± 1.0
Thr^{P6}	-2.2 ± 1.2
Ala^{P3}	-2.2 ± 0.6
Arg^{P5}	-2.1 ± 2.4
Pro^{P4}	-1.6 ± 0.5
Val^{P1}	3.0 ± 1.8

**$COM^{\sim}_{B27}$ ΔG_b -77.8 ± 9.4 [kcal/mol]**	
**Residue**	**ΔG_b total [kcal/mol]**
Leu^{P9}	-6.4 ± 3.1
Leu^{P7}	-5.6 ± 0.8
Thr^{P2}	-5.5 ± 1.8
Leu^{P8}	-5.1 ± 1.1
Arg^{P5}	-2.3 ± 1.8
Ala^{P3}	-2.1 ± 0.6
Thr^{P6}	-2.0 ± 1.2
Pro^{P4}	-1.7 ± 0.5
Val^{P1}	2.4 ± 2.9

**$COM^{\sim}_{Cw7}$ ΔG_b -94.9 ± 8.5 [kcal/mol]**	
**Residue**	**ΔG_b total [kcal/mol]**
Met^{P2}	-9.3 ± 1.1
Leu^{P9}	-8.9 ± 2.8
Leu^{P8}	-5.1 ± 1.0
Ala^{P6}	-5.0 ± 0.6
Leu^{P7}	-5.0 ± 0.7
Arg^{P5}	-4.2 ± 1.9
Ala^{P3}	-3.3 ± 0.8
Pro^{P4}	-2.0 ± 0.6
Val^{P1}	2.7 ± 2.8

**$COM^{-}_{Hsp60sp}$ ΔG_b -82.1 ± 8.3 [kcal/mol]**	
**Residue**	**ΔG_b total [kcal/mol]**
Met^{P2}	-11.9 ± 0.9
Leu^{P9}	-6.7 ± 2.0
Arg^{P3}	-6.3 ± 2.1
Val^{P8}	-3.5 ± 1.2
Val^{P5}	-2.1 ± 1.2
Pro^{P4}	-1.5 ± 0.5
Ser^{P6}	-1.0 ± 1.2
Gln^{P1}	2.1 ± 2.2
Arg^{P7}	3.2 ± 2.3

**$COM^{-}_{B7\_R5V}$ ΔG_b -90.7 ± 7.5 [kcal/mol]**	
**Residue**	**ΔG_b total [kcal/mol]**
Met^{P2}	-10.7 ± 1.7
Leu^{P9}	-8.8 ± 2.8
Leu^{P8}	-4.9 ± 0.9
Val^{P7}	-4.4 ± 0.7
Thr^{P6}	-4.1 ± 0.9
Val^{P5}	-3.1 ± 1.0
Ala^{P3}	-2.9 ± 0.6
Pro^{P4}	-1.5 ± 0.5
Val^{P1}	1.3 ± 2.1

Table S4. Peptide interaction energies, where energy is calculated between nonameric peptide and HLA-E/β2m/NKG2A/CD94 on equilibrated part of the trajectories (last 1 μs of the production run) using the gmx energy module of the Gromacs2016 software package.

	VDW [kJ/mol]	EEL [kJ/mol]	Total E [kJ/mol]	Total E [kcal/mol]
**COM^{+}_{G}**	-718.3 ± 51.5	-384.0 ± 23.4	-1102.3 ± 74.8	-263.3 ± 17.9
**$^{allele}COM^{+}_{G}$**	-763.8 ± 79.7	-371.6 ± 23.4	-1135.3 ± 103.1	-271.2 ± 24.6
**COM^{+}_{B7}**	-665.8 ± 53.8	-346.0 ± 29.2	-1011.8 ± 83.0	-241.7 ± 19.8
**$COM^{-}_{Hsp60sp}$**	-576.7 ± 76.3	-376.9 ± 22.9	-953.6 ± 99.2	-227.8 ± 23.7
**$COM^{-}_{B7\_R5V}$**	-532.8 ± 60.8	-354.9 ± 20.9	-887.7 ± 81.7	-212.0 ± 19.5
**$COM^{\sim}_{B27}$**	-606.4 ± 81.3	-331.0 ± 22.8	-937.4 ± 104.1	-223.9 ± 24.9
**$COM^{\sim}_{Cw7}$**	-663.7 ± 99.7	-335.6 ± 25.6	-999.3 ± 125.2	-238.7 ± 29.9

Table S5. Average distances and stabilities of the top five interactions between the nonameric peptide-CD94 protein and the nonameric peptide-HLA-E protein pair of residues for the **COM^{+}_{G}** and **COM^{+}_{B7}** selected models, obtained by a pairwise decomposition of the binding free energies (ΔGb) between the nonameric peptide and the remaining HLA-E/β2m/NKG2A/CD94 complex calculated by Molecular Mechanics-Generalized Born Surface Area (MM-GBSA) method. Interaction stability is given as a share of the equilibrated part of the trajectory where distances between specified atoms are below 4 Å (hydrogen bonds) or 6 Å (hydrophobic interactions and cation-π interactions).

**COM^{+}_{G}**				**$COM^{-}_{B7\_R5V}$**			
**Atom 1**	**Atom 2**	**Average distance**	**Interaction stability**	**Atom 1**	**Atom 2**	**Average distance**	**Interaction stability**
NE2@Gln112^{CD94}	O@Thr^{P6}	2.9 ± 0.2	100 %	NE2@Gln112^{CD94}	O@Thr^{P6}	2.9 ± 0.4	98 %
NE2@Gln112^{CD94}	CD2@Phe^{P8}	3.8 ± 0.8	98 %	NE2@Gln112^{CD94}	CD1@Leu^{P8}	6.0 ± 0.6	50 %
NH2@Asn160^{CD94}	CE1@Phe^{P8}	4.2 ± 1.0	91 %	ND2@Asn160^{CD94}	CD1@Leu^{P8}	4.4 ± 0.7	96 %
OE1@Gln112^{CD94}	NH2@Arg^{P5}	3.4 ± 0.4	100 %	NE2@Gln112^{CD94}	CG2@Val^{P5}	3.7 ± 0.6	99 %
ND2@Asn158^{CD94}	CD2@Phe^{P8}	5.2 ± 1.1	81 %	NE2@Gln113^{CD94}	CG1@Val^{P5}	5.1 ± 1.5	67 %
OE1@$^{Glu63HLA-E}$	N@Val^{P1}	3.1 ± 0.4	97 %	OE1@Glu63^{HLA-E}	N@Val^{P1}	3.0 ± 0.4	97%
OE1@Glu152^{HLA-E}	NH1@Arg^{P5}	3.4 ± 0.7	76 %	NZ@Lys146^{HLA-E}	O@Leu^{P9}	3.5 ± 1.1	82 %
NZ@Lys146^{HLA-E}	O@Leu^{P9}	3.5 ±1.2	83 %	OG@Ser143^{HLA-E}	OXT@Leu^{P9}	3.1 ± 0.8	81 %
OE1@Gln156^{HLA-E}	NH1@Arg^{P5}	2.9 ± 0.2	99 %	OH@Tyr171^{HLA-E}	N@Val^{P1}	3.1 ± 0.3	98 %
OG@Ser143^{HLA-E}	OXT@Leu^{P9}	3.2 ± 0.9	73 %	OH@Tyr7^{HLA-E}	N@Val^{P1}	3.2 ± 0.4	95 %

Table S6. Molecular mechanics-generalized born surface area (MM-GBSA) binding free energies (ΔG_b) between receptor (NKG2A/CD94) and ligand (HLA-E/β2m/peptide) and its pair-wise decomposition (kcal/mol) calculated over time interval between 600 and 900 ns of the MD trajectory in the production run for **COM$^{+}_{G,}$^{allele}COM$^{+}_{G}$** and **COM$^{+}_{B7}$** models between HLA-E/β2m/peptide and NKG2A/CD94. In the table are listed top 15 interactions with highest energy.

**COM$^{+}_{G}$ ΔG_b -38.9 ± 11.5 [kcal/mol]**		
**Res 1**	**Res 2**	**ΔG_b total**
HLA-E		
Asp69	Arg171^{CD94}	-15.6 ± 1.4
Arg75	Asp163^{CD94}	-13.6 ± 3.9
Asp162	Lys217^{NKG2A}	-11.8 ± 9.8
Asp162	Lys199^{NKG2A}	-9.9 ± 5.0
Arg68	Glu164^{CD94}	-7.4 ± 6.2
Gln72	Glu164^{CD94}	-6.4 ± 2.8
Arg68	Asp168^{CD94}	-5.2 ± 5.1
Asp162	Arg215^{NKG2A}	-4.9 ± 4.6
Asn148	Arg137^{NKG2A}	-4.0 ± 1.7
Arg75	Glu164^{CD94}	-3.8 ± 4.1
His155	Ser172^{NKG2A}	-3.3 ± 0.8
Asp162	Gln212^{NKG2A}	-3.3 ± 2.9
Gln72	Phe114^{CD94}	-2.9 ± 0.4
Peptide		
Thr^{P6}	Gln112^{CD94}	-3.9 ± 0.4
Phe^{P8}	Gln112^{CD94}	-3.1 ± 0.5

**^{alelle}COM$^{+}_{G}$ ΔG_b -41.7 ± 11.0 [kcal/mol]**		
**Res 1**	**Res 2**	**ΔG_b total**
HLA-E		
Asp69	Arg171^{CD94}	-19.4 ± 2.2
Asp162	Lys217^{NKG2A}	-18.2 ± 2.8
Arg75	Asp163^{CD94}	-15.1 ± 2.7
Arg68	Asp168^{CD94}	-10.4 ± 4.4
Asp69	Arg68^{HLA-E}	-7.3 ± 1.2
Asp162	Lys199^{NKG2A}	-6.7 ± 5.9
Gln72	Glu164^{CD94}	-5.9 ± 2.3
Arg68	Gln79^{CD94}	-5.8 ± 1.2
Asp162	Arg215^{NKG2A}	-5.5 ± 3.3
Arg65	Asp168^{CD94}	-3.5 ± 4.8
Ala158	Arg212^{NKG2A}	-3.3 ± 1.6
Glu154	Arg157^{HLA-E}	-3.1 ± 0.7
Peptide		
Thr^{P6}	Gln112^{CD94}	-4.0 ± 0.4
Arg^{P5}	Glu152^{HLA}	-3.4 ± 1.4
NKG2A		
Lys217	Asp200^{NKG2A}	-4.0 ± 0.9

**COM$^{+}_{B7}$ ΔG_b -37.7 ± 13.2 [kcal/mol]**		
**Res 1**	**Res 2**	**ΔG_b total**
HLA-E		
Asp69	Arg171^{CD94}	-20.2 ± 1.9
Arg75	Asp163^{CD94}	-18.2 ± 2.7
Arg68	Asp168^{CD94}	-10.9 ± 3.2
Arg157	Asp200^{NKG2A}	-10.8 ± 9.4
Arg65	Asp168^{CD94}	-10.8 ± 5.2
Asp162	Lys199^{NKG2A}	-8.6 ± 5.0
Asp162	Arg215^{NKG2A}	-7.1 ± 4.0
Arg68	Glu164^{CD94}	-5.9 ± 4.6
Asp69	Arg65^{HLA-E}	-5.6 ± 1.0
Glu154	Lys217^{NKG2A}	-5.4 ± 4.8
Asp149	Arg137^{NKG2A}	-4.3 ± 4.5
Gln72	Glu164^{CD94}	-3.9 ± 2.7
Arg75	Thr146^{CD94}	-2.9 ± 2.0
Peptide		
Arg^{P5}	Glu152^{HLA-E}	-3.2 ± 1.2
Thr^{P6}	Gln112^{CD94}	-2.9 ± 1.0

Table S7. Molecular mechanics-generalized born surface area (MM-GBSA) binding free energies (ΔG_b) receptor (NKG2A/CD94) and ligand (HLA-E/β2m/peptide) and its pair-wise decomposition (kcal/mol) calculated over time interval between 600 and 900 ns of the MD trajectory in the production run for **COM$^-$$_{Hsp60sp}$**, and **COM$^-$$_{B7_R5V}$** between HLA-E/β2m/peptide and NKG2A/CD94. In the table are gathered top 15 interactions.

**COM$^-$$_{Hsp60sp}$ ΔG_b -25.9 ± 12.9 [kcal/mol]**		
**Res 1**	**Res 2**	**ΔG_b total**
HLA-E		
Arg79	Asp163^{CD94}	-16.4 ± 2.6
Asp69	Arg171^{CD94}	-16.4 ± 1.4
Arg75	Glu164^{CD94}	-12.8 ± 6.0
Gln72	Glu164^{CD94}	-6.6 ± 2.1
Arg75	Asp163^{CD94}	-5.9 ± 5.6
Asp162	Lys217^{NKG2A}	-5.9 ± 3.7
Arg68	Asp168^{CD94}	-4.8 ± 4.9
Arg68	Gln79^{CD94}	-3.8 ± 3.3
Asp69	Gln113^{CD94}	-3.2 ± 1.7
Glu152	Gln112^{CD94}	-3.1 ± 2.7
Arg68	Glu164^{CD94}	-2.7 ± 5.2
Gln72	Asn170^{CD94}	-2.6 ± 0.5
Ser151	Arg137^{NKG2A}	-2.6 ± 1.4
Peptide		
NKG2A		
Asp200	Arg215^{NKG2A}	-2.9 ± 1.4
Asp200	Lys217^{NKG2A}	-2.7 ± 1.4

**COM$^-$$_{B7_R5V}$ ΔG_b -43.7 ± 13.9 [kcal/mol]**		
**Res 1**	**Res 2**	**ΔG_b total**
HLA-E		
Asp69	Arg171^{CD94}	-19.6 ± 2.2
Asp162	Lys217^{NKG2A}	-16.0 ± 3.9
Glu154	Arg137^{NKG2A}	-11.8 ± 6.4
Asp162	Lys199^{NKG2A}	-11.2 ± 3.9
Arg75	Glu164^{CD94}	-10.0 ± 6.1
Arg68	Gln79^{CD94}	-7.5 ± 1.3
Arg75	Asp163^{CD94}	-7.4 ± 5.6
Glu166	Arg215^{NKG2A}	-7.2 ± 6.4
Gln72	Glu164^{CD94}	-6.6 ± 2.3
Glu154	Arg131^{HLA-E}	-6.3 ± 2.1
Asp69	Arg68^{HLA-E}	-6.2 ± 0.8
Asp162	Gln212^{NKG2A}	-5.8 ± 1.3
Arg131	Ser223^{NKG2A}	-4.7 ± 1.3
Glu154	Arg157^{HLA-E}	-4.0 ± 1.0
Peptide		
Thr^{P6}	Gln112^{CD94}	-3.6 ± 0.5
NKG2A		
Asp200	Lys217^{NKG2A}	-2.7 ± 1.4

Table S8. Molecular mechanics-generalized born surface area (MM-GBSA) binding free energies (ΔG_b) between receptor (NKG2A/CD94) and ligand (HLA-E/β2m/peptide) and its per-residue decomposition (kcal/mol) calculated over time interval between 600 and 900 ns of the MD trajectory in the production run for **COM$^{+}_{G}$**, **^{allele}COM$^{+}_{G}$**, **COM$^{+}_{B7}$**, **COM$^{-}_{Hsp60sp}$**, and **COM$^{-}_{B7_R5V}$**. In the table are gathered first 10 ligand and receptor residues with the most favorable binding free energies.

**COM$^{+}_{G}$ ΔG_b -38.9 ± 11.5 [kcal/mol]**		**^{allele}COM$^{+}_{G}$ ΔG_b -41.7 ± 11.0 [kcal/mol]**		**COM$^{+}_{B7}$ ΔG_b -37.7 ± 13.2 [kcal/mol]**	
**Residue**	**ΔG_b total**	**Residue**	**ΔG_b total**	**Residue**	**ΔG_b total**
Asp162^{HLA-E}	-7.8 ± 5.1	Asp69^{HLA-E}	-12.0 ± 2.3	Asp69^{HLA-E}	-12.0 ± 2.0
Asp69^{HLA-E}	-5.1 ± 1.1	Asp162^{HLA-E}	-5.4 ± 3.3	Arg75^{HLA-E}	-5.8 ± 2.1
Arg68^{HLA-E}	-4.6 ± 2.0	Arg75^{HLA-E}	-4.0 ± 1.9	Asp162^{HLA-E}	-4.0 ± 3.1
Gln72^{HLA-E}	-3.7 ± 1.9	Ala158^{HLA-E}	-2.9 ± 1.2	Arg68^{HLA-E}	-3.0 ± 3.7
Arg75^{HLA-E}	-3.5 ± 1.8	Glu152^{HLA-E}	-1.8 ± 1.4	Glu154^{HLA-E}	-2.3 ± 2.3
Arg65^{HLA-E}	-3.0 ± 2.1	Glu154^{HLA-E}	-1.7 ± 1.5	Ile73^{HLA-E}	-2.0 ± 0.5
His155^{HLA-E}	-2.3 ± 0.8	Gln72^{HLA-E}	-1.4 ± 1.6	Arg62^{HLA-E}	-1.9 ± 3.0
Asn148^{HLA-E}	-1.7 ± 1.0	Ile73^{HLA-E}	-1.3 ± 0.4	Glu152^{HLA-E}	-1.8 ± 1.2
Phe^{P8}	-5.5 ± 1.0	Phe^{P8}	-4.6 ± 1.0	Gln72^{HLA-E}	-1.5 ± 2.2
Thr^{P6}	-2.3 ± 0.5	Thr^{P6}	-2.4 ± 0.5	His155^{HLA-E}	-1.4 ± 1.0
Arg171^{CD94}	-5.3 ± 0.8	Phe114^{CD94}	-5.9 ± 0.7	Asp163^{CD94}	-6.1 ± 1.9
Phe114^{CD94}	-4.6 ± 0.5	Asp163^{CD94}	-5.2 ± 1.4	Asp168^{CD94}	-5.8 ± 2.8
Asp163^{CD94}	-4.4 ± 1.7	Gln79^{CD94}	-2.9 ± 1.2	Phe114^{CD94}	-4.4 ± 0.7
Glu164^{CD94}	-2.6 ± 3.9	Asp168^{CD94}	-1.9 ± 2.3	Leu162^{CD94}	-2.0 ± 0.6
Leu162^{CD94}	-2.1 ± 0.5	Ser110^{CD94}	-1.7 ± 1.2	Thr146^{CD94}	-1.8 ±1.5
Pro171^{NKG2A}	-1.8 ± 0.4	Leu162^{CD94}	-1.7 ± 0.7	Arg171^{CD94}	-1.8 ± 1.6
Arg137^{NKG2A}	-1.6 ± 1.3	Lys217^{NKG2A}	-8.2 ± 3.4	Ser110^{CD94}	-1.0 ± 1.7
Ile226^{NKG2A}	-1.6 ± 0.3	Pro171^{NKG2A}	-2.4 ± 0.5	Asp200^{NKG2A}	-3.8 ± 4.6
Gln212^{NKG2A}	-1.4 ± 1.6	Ile225^{NKG2A}	-2.0 ± 0.4	Pro171^{NKG2A}	-2.2 ± 0.5
Ser172^{NKG2A}	-1.4 ± 0.7	Ile226^{NKG2A}	-2.0 ± 0.3	Arg137^{NKG2A}	-1.6 ± 2.4

**COM$^{-}_{Hsp60sp}$ ΔG_b -25.9 ± 12.9 [kcal/mol]**		**COM$^{-}_{B7_R5V}$ ΔG_b -43.7 ± 13.9 [kcal/mol]**	
**Residue**	**ΔG_b total**	**Residue**	**ΔG_b total**
Asp69^{HLA-E}	-5.7 ± 1.3	Asp69^{HLA-E}	-12.3 ± 2.3
Arg79^{HLA-E}	-4.4 ± 1.3	Glu154^{HLA-E}	-9.3 ± 3.9
Gln72^{HLA-E}	-3.6 ±1.3	Asp162^{HLA-E}	-8.1 ± 2.7
Arg65^{HLA-E}	-2.9 ± 2.8	Gln72^{HLA-E}	-3.7 ± 1.8
Arg75^{HLA-E}	-2.5 ± 2.0	Arg75^{HLA-E}	-3.5 ± 1.7
Glu166^{HLA-E}	-2.0 ± 1.9	His155^{HLA-E}	-2.1 ± 0.6
Asp162^{HLA-E}	-1.8 ± 1.6	Ile73^{HLA-E}	-1.8 ± 0.4
Arg68^{HLA-E}	-1.7 ± 3.2	Glu166^{HLA-E}	-1.7 ± 2.4
Val76^{HLA-E}	-1.6 ± 0.5	Thr^{P6}	-2.3 ± 0.5
Ile73^{HLA-E}	-1.6 ± 0.5	Leu^{P8}	-2.2 ± 0.4
Asp163^{CD94}	-8.4 ± 4.0	Phe114^{CD94}	-5.2 ± 0.6
Glu164^{CD94}	-6.2 ± 3.2	Glu164^{CD94}	-4.0 ± 2.9
Arg171^{CD94}	-4.8 ± 0.8	Gln79^{CD94}	-3.6 ± 1.3
Phe114^{CD94}	-4.4 ± 0.7	Asp63^{CD94}	-3.3 ± 4.4
Thr146^{CD94}	-2.8 ± 1.3	Leu162^{CD94}	-2.5 ± 0.8
Leu162^{CD94}	-1.9 ±0.8	Ser223^{NKG2A}	-3.1 ± 1.3
Asp200^{NKG2A}	-2.1 ± 2.3	Ile225^{NKG2A}	-2.1 ± 0.6
Pro171^{NKG2A}	-1.6 ± 0.5	Ile226^{NKG2A}	-2.1 ± 0.5
Ile226^{NKG2A}	-1.5 ± 0.6	Arg215^{NG2A}	-2.1 ± 3.2
Ile225^{NKG2A}	-1.2 ± 0.6	Arg137^{NKG2A}	-1.9 ± 2.1

Supporting Movies

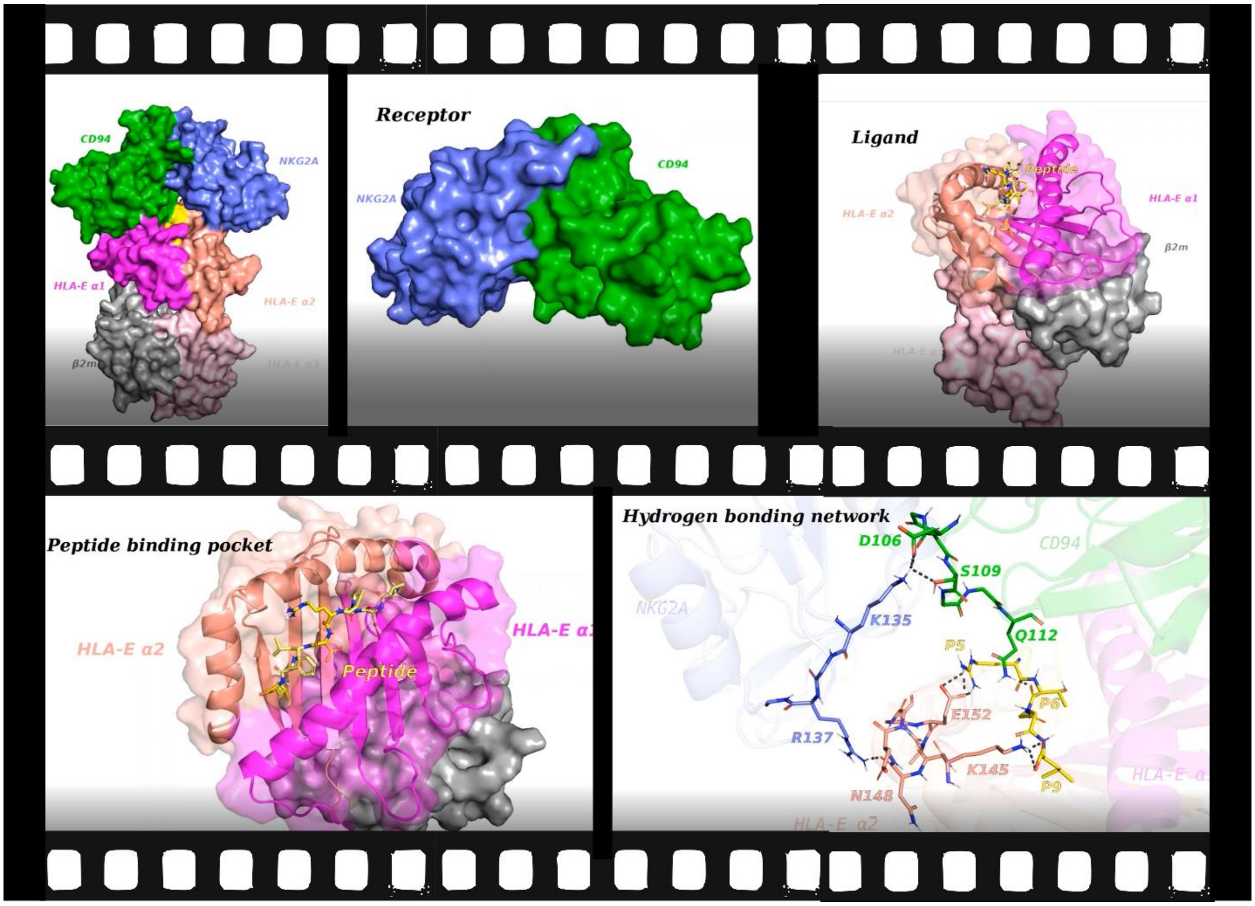

Movie S1. The presentation of the immune complex HLA-E/β2m/peptide/NKG2A/CD94, receptor and ligand part, placement of the nonameric peptide between HLA-E α1 and 2 domains, and identified key signaling networks.
